# A molecular switch for coordinating kinesin and dynein transport of mitochondrial cargo

**DOI:** 10.1126/science.aeh1475

**Published:** 2026-08-06

**Authors:** Christina Gladkova, Maria G. Paez-Segala, William P. Grant, Mark Kittisopikul, Samuel A. Myers, Yuxiao Wang, Ronald D. Vale

**Affiliations:** 1 Janelia Research Campus, Howard Hughes Medical Institute, Ashburn, VA, USA; 2 Shrike Research, New York, NY, USA; 3 La Jolla Institute for Immunology, La Jolla, CA, USA; 4 Department of Cellular and Molecular Pharmacology, University of California, San Francisco, San Francisco, CA, USA

## Abstract

The cellular distribution of mitochondria in response to stress and local energy needs is governed by the relative activities of the microtubule-based molecular motors kinesin and dynein. The mechanism for switching between these two opposite polarity microtubule motors remains unknown. Here, we coupled a cellular synthetic cargo transport assay with AlphaFold2-guided mutagenesis to identify a regulatory helix in the mitochondrial adaptor protein (TRAK) that mediates switching between kinesin- and dynein-driven transport. Differences in the helix sequence explained why two near-identical TRAK isoforms transported mitochondria in predominantly opposite directions. Phosphorylation of the regulatory helix by stress-activated kinases caused the activation of dynein and dissociation of kinesin. Our results reveal a molecular mechanism for coordinating the directional transport of mitochondria in response to intracellular signals.

## Main Text:

Membrane-bound organelles are transported by kinesins and cytoplasmic dynein along microtubules. In most cells microtubules are organized with their minus ends anchored at the centrosome near the cell center and plus ends oriented towards the cell periphery. Most organelles exhibit bidirectional transport, with episodes of kinesin-driven (plus-end-directed; anterograde) and dynein-driven (minus-end-directed; retrograde) movement (1). The distribution of a particular organelle is governed by the net activity of the two motors. Organelles such as mitochondria tend to move in the anterograde or retrograde direction at speeds characteristic of the maximal velocity of kinesin or dynein motors (2–4). Kinesin and dynein are thus not continuously opposing one another and mechanisms for coordinating motor binding and/or activity likely exist.

**Fig. 1.**
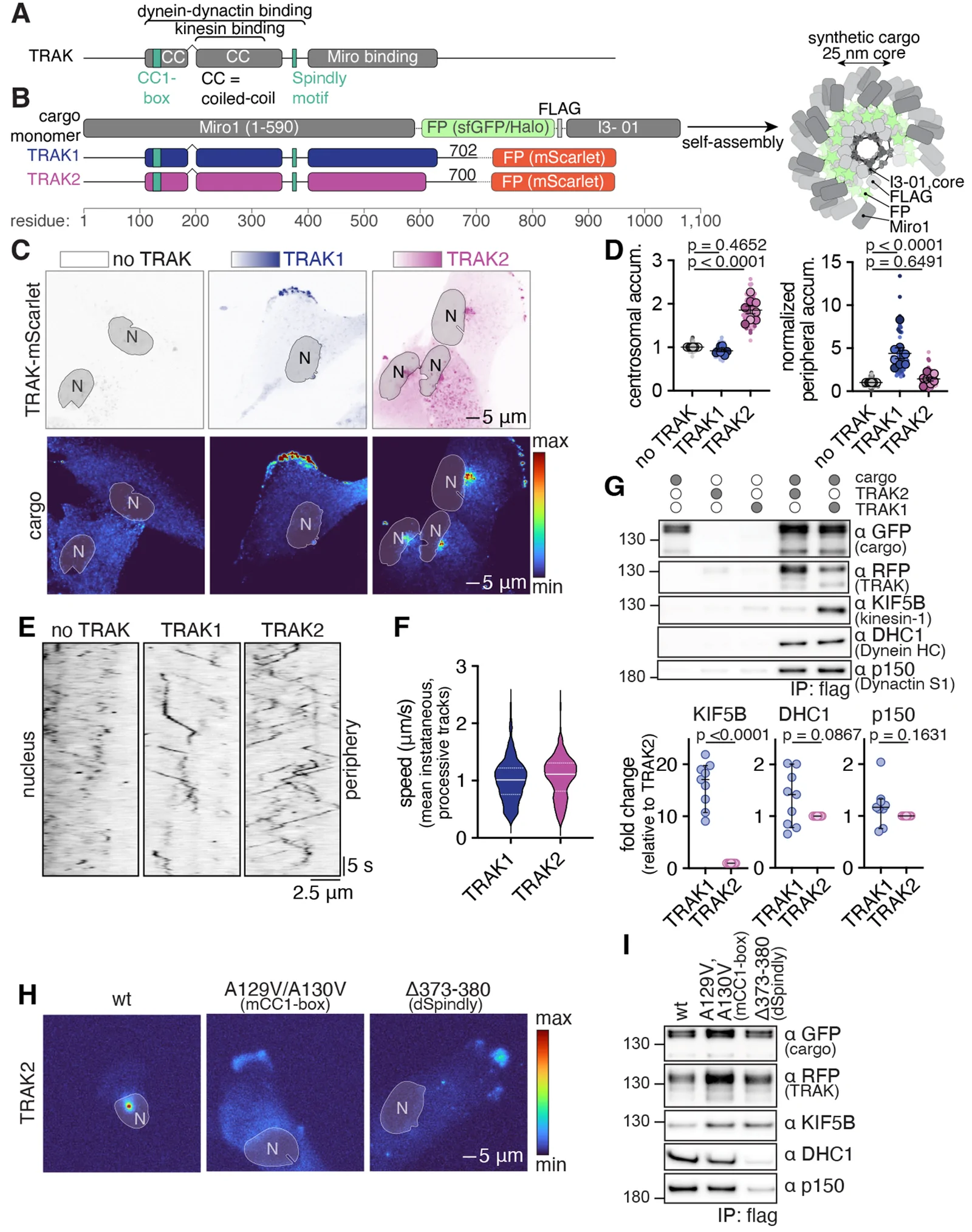
Microtubule-based transport of synthetic mitochondrial cargo. (**A**) Common domain organization for TRAK1 and TRAK2 with interaction sites annotated based on prior work. (**B**) Generating synthetic mitochondrial cargo by lentiviral expression of Miro1/FP/FLAG/I3-01 (FP = Superfolder GFP (sfGFP), or a Halo-Janelia Fluor Dye conjugate) and TRAK/mScarlet fusion proteins in hTERT RPE-1 cells. 60 copies of the I3-01 fusion assemble to form the synthetic cargo (35). (**C**) Representative maximum intensity projection images of cells expressing TRAK (top) and synthetic cargo (bottom). (**D**) Phenotype quantification of synthetic cargo accumulation from cells as in (C) imaged at lower resolution. Data points correspond to normalized median values calculated for a field of view with ~100 cells, summary statistics (mean, SEM) and color-matched means from repeated experiments are superimposed. See fig. S2 and Methods for analysis description. Nested ANOVA with Dunnett’s test (n = 8), p-values indicated in the figure. (**E**) Kymographs showing processive motility of PA-JF646 labelled synthetic cargo after photoactivation. Kymographs were generated by reslicing time series shown in Movie S2 as indicated in fig. S3C. (**F**) Mean instantaneous speed averaged over the length of processive tracks (maximum distance travelled >3.0 μm). This speed was 1.02 ± 0.02 μm/s for TRAK1 (n = 21 cells, 573 tracks), and 1.07 ± 0.02 μm/s for TRAK2 (n = 47 cells, 270 tracks). See fig. S3C–F and Methods for analysis description. (**G**) Top: Immunoblot analysis of the motor-adaptor components associated with FLAG-purified cargo from indicated cell lines. Bottom: quantification of relative motor protein levels normalized to TRAK2. One-sample t-tests (n = 9), p-values indicated in the figure. Error bars show the median and 95% confidence interval. (**H**) Representative maximum intensity projection images of cargo accumulation from indicated cell lines. See fig. S4F for quantification. (**I**) Immunoblot analysis of the motor-adaptor components associated with FLAG-purified cargo from indicated cell lines. See fig. S4G for quantification.

Mitochondria constitute perhaps the best studied example of bidirectional organelle transport (5, 6). Mitochondrial transport by kinesin-1 supports energetically-demanding peripheral processes such as cell outgrowth or synaptic activity (7, 8). Dynein based transport towards the centrosome is necessary for turnover of damaged mitochondria (9). TRAK, a multi-functional motor adaptor protein (10), recruits kinesin-1 and dynein (11–13) and binds to mitochondria via multiple interaction partners that include the outer membrane receptor Miro (13–16). TRAK interacts with dynein through motifs that are conserved in related adaptor proteins (Fig. 1A) (17): A CC1-box that interacts with dynein’s light intermediate chain (LIC) (18, 19), a Spindly motif that binds dynactin, a dynein activator (20), and a long coiled-coil domain that is sandwiched between dynein and dynactin in the active motor complex (17–19). The TRAK coiled-coil also binds kinesin-1 directly (21–23). Recent in vitro studies have examined how motor transport is coordinated by TRAK, but emerged with two different models, suggesting either co-dependence (24) (one motor aiding the other), or mutual exclusivity (25) of kinesin and dynein activity.

Vertebrates have two TRAK adaptor genes, TRAK1 and TRAK2, that are ~75% similar in their motor binding regions (fig. S1). Both TRAKs bind the dynein-dynactin complex equally well; however, TRAK1’s affinity for kinesin is much greater compared to TRAK2 (11, 25). In hippocampal neurons, TRAK1 preferentially transports cargos into the axon, while TRAK2 predominantly drives transport into dendrites (11, 26). Previous cellular studies also found that TRAK-mediated transport is modulated in response to different cues such as cell cycle status (27), glucose availability (28, 29), reactive oxygen species (ROS) (30), and ATP/AMP ratio (31). However, the differences between the two TRAKs, how TRAK switches between kinesin- and dynein-driven transport, and how motor switching can be influenced by intracellular signals remain poorly understood.

## A synthetic cargo recapitulates motor coordination by TRAK

The complex interactions of mitochondria with actin, microtubules, and other organelles, as well as their fission and fusion dynamics, pose challenges for isolating and studying the coordination of microtubule-based motors in cells (5, 32, 33). Previous studies have mistargeted adaptor proteins to peroxisomes to mitigate some of these issues (11, 32, 34), but such assays still involve a complex organelle with endogenous motors and regulatory mechanisms. Instead, we created a synthetic cargo using an engineered protein (I3-01) that self-assembles into ~25 nm dodecahedral nanocages (Fig. 1B) (35). To couple these nanocages to the microtubule-based mitochondrial transport machinery, I3-01 was fused to the cytoplasmic domain of Miro1, a mitochondrial receptor for TRAK. To visualize or immunoprecipitate nanocages, fluorescent and FLAG tags were fused to I3-01, respectively. The steady-state distributions of synthetic cargo in the perinuclear and peripheral regions were measured in hundreds of cells using a computational pipeline (fig. S2) (36).

When expressed in hTERT RPE-1 cells, sfGFP-labelled synthetic cargos were uniformly distributed throughout the cytoplasm, suggesting that endogenous TRAK is insufficient for their robust transport (Fig. 1C; fig. S3A). However, co-expression of TRAK1 or TRAK2 led to microtubule-dependent relocalization of synthetic cargo replicating prior findings (37) (Fig. 1C,D; fig. S3B, Movie S1). Co-expression of a TRAK1 construct resulted in the peripheral accumulation of synthetic cargo (Fig. 1C,D), consistent with kinesin-driven transport. On the other hand, co-expression of a TRAK2 construct resulted in predominant accumulation of synthetic cargo at the perinuclear region (Fig. 1C,D), consistent with dynein-driven transport. For TRAK2, some cells exhibited both perinuclear and peripheral accumulation (Fig. 1C), indicative of bidirectional transport, although peripheral accumulation was not statistically significant at the population level (Fig. 1D). Cargoes labelled with a photoactivatable JF-646 dye conjugated to HaloTag (38) revealed directional transport at rates similar to those reported for kinesin- and dynein-driven transport of mitochondria in vivo (2, 39) (Fig. 1E,F; fig. S3C-F, Movie S2). Consistent with previous studies (11, 25), kinesin was ~20-fold enriched in TRAK1-versus TRAK2-driven synthetic cargo, while dynein and dynactin levels were comparable in both (Fig. 1G).

Next, we investigated how motor protein depletion by RNAi affected synthetic cargo localization. As expected, dynein knockdown resulted in loss of perinuclear accumulation for TRAK2 (fig. S4A), and kinesin knockdown eliminated peripheral accumulation for both TRAK1 and TRAK2 (fig. S4B,C). Dominant cargo accumulation was unaffected by the knockdown of the opposing motors (kinesin for TRAK2 and dynein for TRAK1) (fig. S4C,D). For TRAK2 with dynein RNAi, we also observed a relocalization of cargo to the periphery, revealing that kinesin can become the dominant motor in the absence of dynein. However, in the converse scenario for TRAK1 and kinesin RNAi, cargo did not relocalize to the perinuclear area, indicating that dynein could not take over transport in the absence of kinesin, even though it remained TRAK-associated (fig. S4E). This finding is consistent with in vitro motility studies showing that anterograde-moving TRAK complexes contain both kinesin and inactive dynein-dynactin (25). Together, these findings establish TRAK2 as a bidirectional adaptor and suggest that TRAK1 carries the dynein-dynactin complex as inactive cargo.

To dissect the requirements for TRAK-mediated dynein activation, we mutated the conserved CC1-box and Spindly motifs that interact with dynein LIC and dynactin respectively. For TRAK2, both mutants reduced dynein activity and switched cargo transport from bidirectional to predominantly kinesin-driven (Fig. 1H, fig. S4F). However, although the Spindly motif mutant diminished interaction of TRAK2 with dynein and dynactin, both motor components remained associated with the CC1-box mutant (Fig. 1I, fig. S4G). Similarly, for TRAK1, dynein-dynactin association was abolished by the Spindly motif mutant but not the CC1-box mutant (fig. S4H). This result indicates that dynein-dynactin bound to TRAK through the Spindly motif alone is inactive.

In summary, our synthetic cargo assay showed that TRAK2 can switch between kinesin- and dynein-mediated transport; dynein activity is normally dominant, but kinesin takes over when dynein activity is reduced. In contrast, TRAK1 is a kinesin transporter that carries dynein-dynactin as inactive cargo. Our results further indicate that the Spindly motif in TRAK adaptors is required for dynein-dynactin association and that additional binding of the LIC to the CC1-box is needed for activation of dynein motility.

## AlphaFold insight into TRAK-mediated motor coordination

**Fig. 2.**
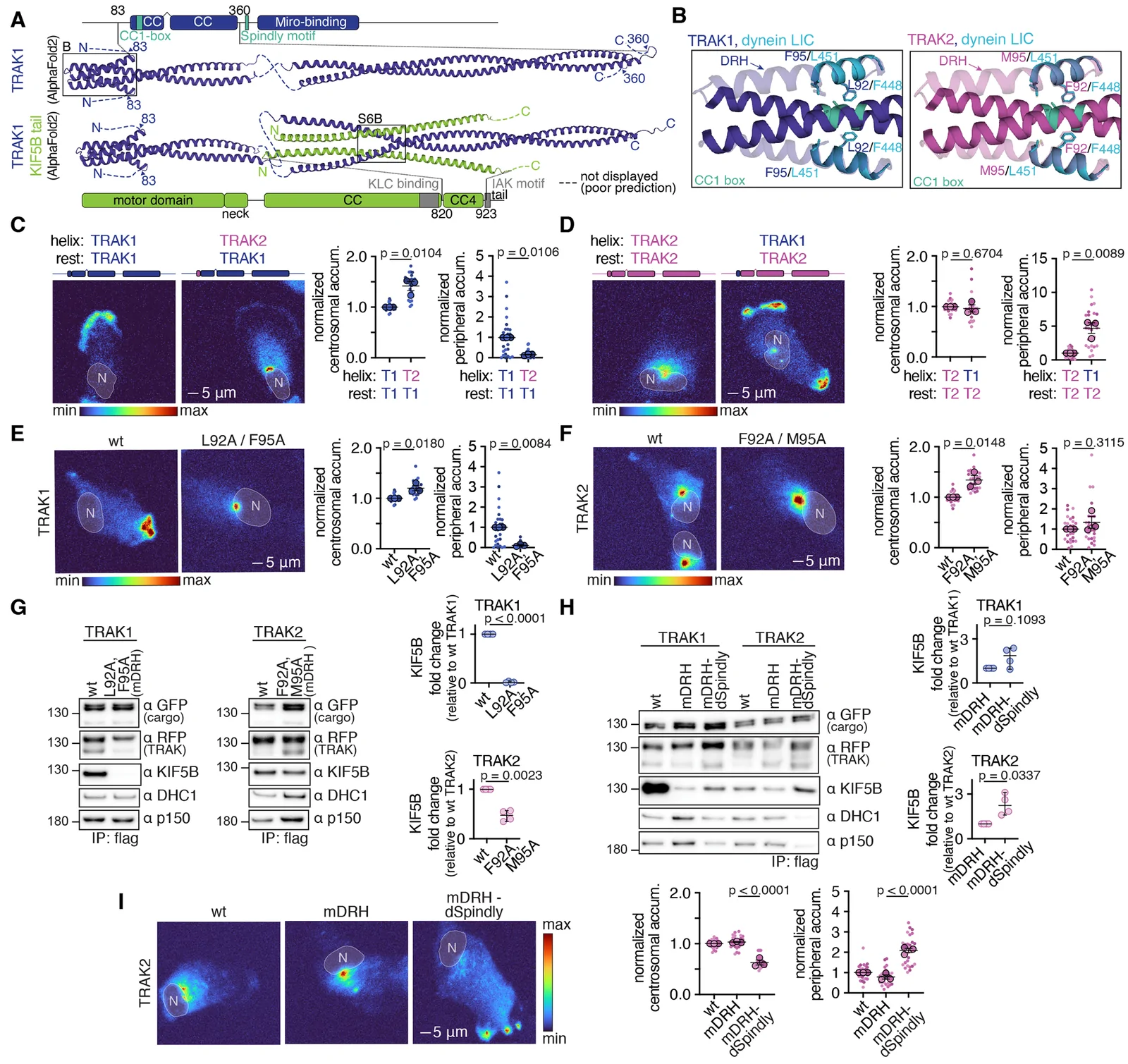
An AlphaFold2-derived model for TRAK-motor interactions. (**A**) AlphaFold2 predictions for TRAK1 (blue). Top: N-terminal coiled-coil dimer (residues shown 83-360). Bottom: N-terminal coiled-coil dimer in complex with the CC4 region of kinesin-1 (green, residues shown 820-923). For clarity, the TRAK1 coiled-coil is split into two separately aligned sections at a site of disorder (see fig. S5). (**B**) Inset from the TRAK1 (blue, left) and TRAK2 (purple, right) coiled-coil prediction highlighting the CC1-box (cyan) with dynein LIC (light blue) superimposed based on a crystal structure of the dynein LIC:BICD2 adaptor complex (18) (PDB ID: 6pse). Hydrophobic residues of the N-terminal folded-back helix (F95, L92 in TRAK1 or M95, F92 in TRAK2) mimic interfacing dynein LIC residues (F448, F451). (**C**), (**D**), (**E**), (**F**) Representative maximum intensity projection images and quantification of cargo accumulation from indicated cell lines. Nested t-test (n=3), p-values indicated in the figure. Error bars show the mean and SEM. (**G**), (**H**) Left: Immunoblot analysis of the motor-adaptor components associated with FLAG-purified cargo from indicated cell lines. Right: quantification of relative motor protein levels normalized to the DRH mut condition. One-sample t-tests (n=4), p-values indicated in the figure. Error bars show the median and 95% confidence interval. (**I**) Representative maximum intensity projection images and quantification of cargo accumulation from indicated cell lines. Nested ANOVA with Šídák’s test (n=3), p-values indicated in the figure. Error bars show the mean and SEM.

To better understand motor binding to TRAK, we turned to AlphaFold2-multimer (Fig. 2A, fig. S5) (40, 41). AlphaFold2 predicted that the kinesin C-terminal coiled-coil (CC4, aa 820-923) forms a four-helical bundle with the C-terminal portion of the TRAK coiled-coil (aa 205-260) (Fig. 2A, fig. S5). This model is consistent with previous mapping experiments (11, 21) and our finding that an arginine substitution of the conserved E242, which AlphaFold2 predicts forms a buried salt-bridge with kinesin (R866) (fig. S6A,B), modestly decreased kinesin association with the TRAKs and kinesin transport of synthetic cargos (fig. S6C–E).

AlphaFold2 also predicted that an N-terminal TRAK helix (aa 85-106) folded back and interacted with the CC1-box region of the TRAK coiled-coil to form a four-helix bundle. This interaction drew our attention, because it mimics the hydrophobic interface that the dynein LIC forms with the CC1-box region of the BICD2 dynein adaptor in a crystal structure (18) (Fig. 2B). To test whether the N-terminal helix might be involved in motor coordination, we generated chimeric proteins in which the helix region (aa 81-106) was swapped between TRAK1 and TRAK2. Notably, the transport direction of these chimeric proteins was determined by the source (TRAK1 or TRAK2) of the folded-back helix, rather than the bulk of the parent polypeptide (Fig. 2C,D). Despite a large increase of kinesin activity when the TRAK2 helix region was swapped with the TRAK1 sequence (Fig. 2D), kinesin association was not restored to wild-type TRAK1 levels (fig. S7A). This chimera result suggests that the folded-back helix primarily affects dynein activity rather than kinesin binding.

To examine how the N-terminal helix regulates motor activity, we generated alanine substitutions for two key hydrophobic residues (F95, L92 in TRAK1 or M95, F92 in TRAK2) that mimic dynein LIC residues L451 and F448 that interact with the CC1-box. AlphaFold2 predicts that these mutations weaken interactions between the fold-back helix and the CC1-box (fig. S7B), and thus might allow dynein LIC to bind and activate dynein transport. Indeed, the mutant switched cargo accumulation from the periphery to the perinuclear region in TRAK1 (Fig. 2E) and maintained perinuclear accumulation in TRAK2 (Fig. 2F). These results suggest that dynein activation requires a structural rearrangement of the folded-back helix from a “closed” to an “open” conformation that permits dynein LIC binding to the CC1-box. Because of its role in regulating dynein activity, we name this region the “dynein regulatory helix (DRH)”.

The DRH mutant also decreased kinesin binding to both TRAKs (Fig. 2G), raising the possibility that dynein activation following helix opening might disfavour kinesin association. To test this, we introduced an additional Spindly box mutation to DRH mutant TRAK that abolishes the association of dynein-dynactin (Fig. 1I, fig. S4G, H). In TRAK2, the DRH-Spindly double mutant resulted in more kinesin binding (Fig. 2H) and transport (Fig. 2I) compared with the DRH mutant, consistent with the idea that dynein-dynactin activation antagonizes kinesin binding (25). In TRAK1, the DRH-Spindly double mutant had a weak effect on kinesin transport (fig. S7C) and modestly increased kinesin binding compared to DRH alone. Similar to the findings described earlier with the chimeric TRAK1-TRAK2 protein (fig. S7A), the DRH-Spindly double mutant did not restore kinesin association to the wildtype TRAK1 levels (Fig. 2H). Both results highlight an interesting difference in kinesin binding between TRAK1 and TRAK2, and we hypothesize that the closed conformation of the DRH may serve an additional role in promoting kinesin binding to TRAK1. Indeed, a previous study of the TRAK1:KIF5B complex using cross-linking mass spectrometry detected cross-links that placed residues in the four helical bundle (K107 and K138) within 24-30 Å of kinesin K801 (22).

To ascertain whether the DRH motor switching mechanism by TRAK involves the Miro1 mitochondrial receptor, we eliminated Miro1 and linked TRAK directly to the synthetic cargo using the rapamycin-inducible FRB-FKBP interaction (fig. S8A). We found that the directionality of transport similarly depended on DRH helix identity (TRAK1 versus TRAK2; fig. S8B), and the DRH disrupting mutants (fig. S8C). Thus, this switching mechanism for motor directionality resides solely within TRAK and does not require Miro.

Together, our AlphaFold-guided mutagenesis results reveal that dynein activation requires a rearrangement of the folded-back helix from a “closed” to an “open” conformation that permits dynein LIC binding to the CC1-box, activating dynein and favoring kinesin dissociation.

## Stress-induced phosphorylation of TRAK2 activates dynein

**Fig. 3.**
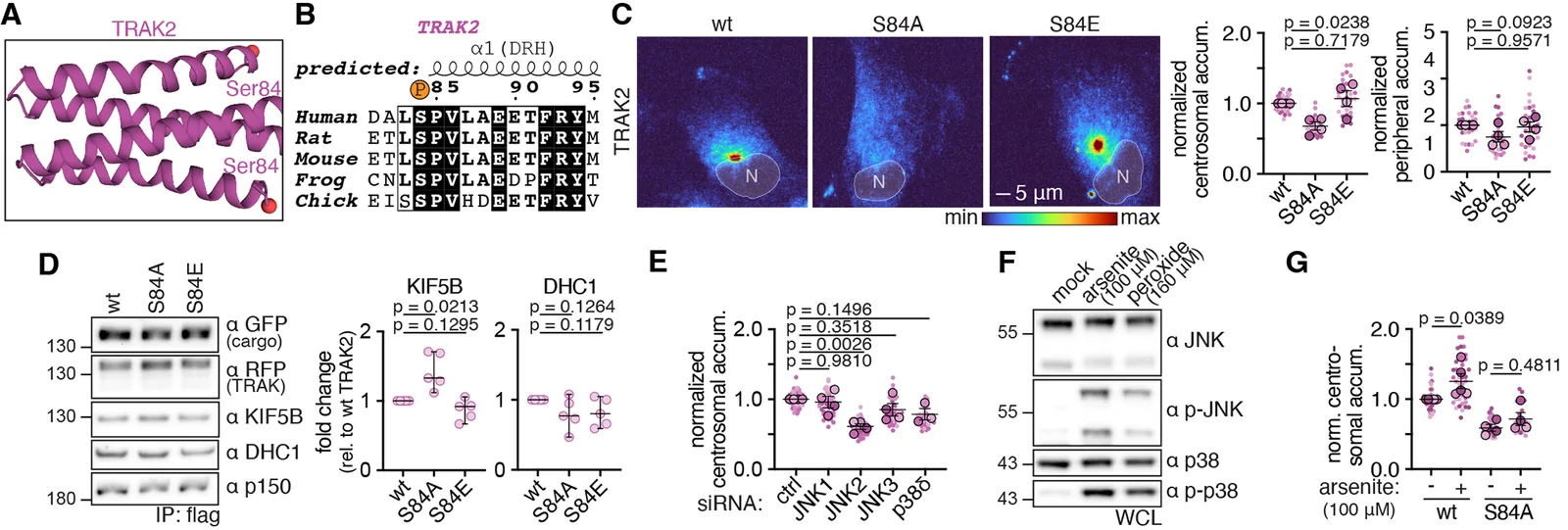
Activation of dynein by stress-induced phosphorylation of TRAK2. (**A**) The TRAK2 S84 phosphorylation site identified by mass spectrometry highlighted in the TRAK2 coiled-coil AlphaFold2 prediction shown as in Fig. 2B. (**B**) S84 conservation in TRAK2. (**C**) Representative maximum intensity projection images and quantification of cargo accumulation from indicated cell lines. Nested ANOVA with Dunnett’s test (n=4), p-values indicated in the figure. Error bars show the mean and SEM. (**D**) Left: Immunoblot analysis of the motor-adaptor components associated with FLAG-purified cargo from indicated cell lines. Right: quantification of relative motor protein levels normalized to wt. One-sample t-tests (n=5), p-values indicated in the figure. Error bars show the median and 95% confidence interval. (**E**) Quantification of cargo accumulation from indicated cell lines. Nested ANOVA with Dunnett’s test (n=4), p-values indicated in the figure. Error bars show the mean and SEM. Representative maximum intensity projection images shown in fig. S10B. (**F**) Immunoblot analysis of untransduced whole cell lysate showing JNK- and p38-kinase activation in response to 3h oxidative treatments as indicated. (**G**) Quantification of cargo accumulation from indicated conditions. Nested ANOVA with Šídák’s test (n=5 (wt), n=4 (S84A)), p-values indicated in the figure. Error bars show the mean and SEM. Representative maximum intensity projection images shown in fig. S10C.

The dynein activation by DRH mutants raised the question of whether the DRH might be subject to physiological regulation. To explore this possibility, we used mass spectrometry to identify post-translational modification sites on TRAK co-immunoprecipitated with our synthetic cargo (fig. S9A–D). Notable among the post-translationally modified sites in TRAK2 was S84, a conserved residue that immediately precedes the DRH (Fig. 3A,B, fig. S9E). In TRAK1, the equivalent T84 residue was not detected as phosphorylated in our mass spectrometry data from RPE-1 cells (fig. S9D).

To examine the role of phosphorylation of S84 in TRAK2-mediated transport, we generated an S84A mutant to block phosphorylation and a negatively-charged S84E mutant as a potential phosphomimetic. AlphaFold2 modelling showed that an S84E substitution lowers the prediction confidence of the DRH closed conformation (fig. S9F). In the synthetic cargo assay, TRAK2 S84A reduced dynein transport towards the centrosome compared with wild-type TRAK2 and the S84E mutant (Fig. 3C). The S84A mutant also bound more kinesin compared to wild-type TRAK2 or the S84E mutant (Fig. 3D), although this effect did not translate into increased particle accumulation at the periphery (Fig. 3C). In contrast, neither the T84E nor T84A mutant significantly altered cargo localization of TRAK1 (fig. S9G), suggesting that T84 phosphorylation does not modulate TRAK1-mediated transport.

The sequence surrounding S84 matched a consensus site for stress-activated MAP kinases (SAPK) JNK1-3 and p38δ (42). We tested whether this class of kinases is involved in activating TRAK2-mediated dynein activity using RNAi knockdown. Of several stress-activated kinases tested, JNK2 depletion caused the strongest reduction in centrosomal accumulation (Fig. 3E, fig. S10A,B).

We next tested whether oxidative stress activates dynein motility (30, 43). Arsenite, an inducer of oxidative stress, activated JNK and p38 MAP kinases (Fig. 3F), and caused perinuclear clustering of TRAK2-driven synthetic cargo (Fig. 3G, fig. S10C). In contrast, TRAK2 S84A mutant cargo showed no significant increase in dynein transport in response to oxidative stress (Fig. 3G, fig. S10C). Together, these results suggest that oxidative stress activates dynein transport through DRH phosphorylation.

## Regulation of mitochondrial transport

**Fig. 4.**
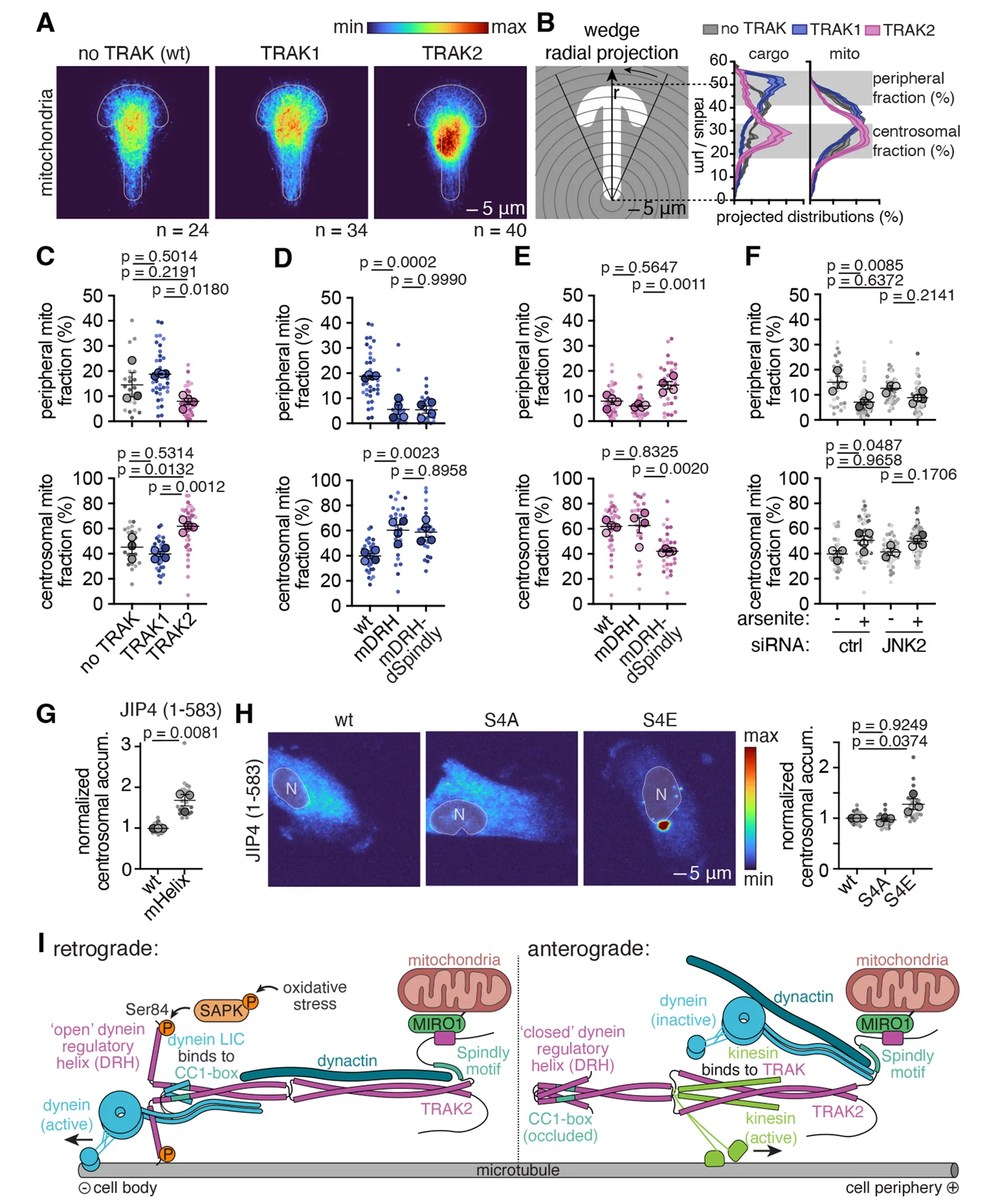
TRAK-mediated dynein regulation shapes the mitochondrial distribution. (**A**) Mean projection images showing mitochondrial distribution in all cells measured for indicated cell lines expressing full length TRAK/mScarlet fusions. To normalize display settings across conditions, summed pixel values were divided by the number of cells measured indicated in the figure. The contour of the template-matched fibronectin pattern is overlaid. (**B**) Right: Schematic showing patterned cell image analysis using a radial projection of signal intensity onto a vertical vector in 1 μm thick radial bins. See fig. S11A and Methods for full analysis description. Left: Projected intensity distributions of synthetic cargo (fig. S11B) and mitochondria (A). The proximal highlighted bin where synthetic cargo accumulates in the presence of TRAK2 overlaps with the microtubule organizing center and we refer to this as the centrosomal bin. The distal highlighted bin where synthetic cargo accumulates in the presence of TRAK1 is referred to as the peripheral bin. (**C**), (**D**), (**E**), (**F**) Quantification of the peripheral and centrosomal mitochondrial fraction from indicated cell lines. Nested ANOVA with Šídák’s test (n=4 except for no TRAK, ctrl siRNA(-Ars) and JNK2 siRNA(-Ars) where n=3), p-values indicated in the figure. Error bars show the mean and SEM. (**G**) Quantification of the accumulation of cargo linked to indicated JIP4 constructs via the FKBP-FRB inducible dimerization system. Nested t-test (n=3), p-values indicated in the figure. Error bars show the mean and SEM. Representative maximum intensity projection images shown in fig. S13C. (**H**) Representative images and quantification of the accumulation of cargo linked to indicated JIP4 constructs as in (G). (**I**) Model for directional switching of mitochondria dictated by the TRAK DRH conformation (see main text for more details).

To study TRAK-mediated motor regulation of native organelles, we measured mitochondrial positioning in cells grown on a patterned fibronectin substrate (fig. S11A) (44). The uniform shape and polarity of these cells provided a reproducible assay for quantitating peripheral versus centrosomal localization (Fig. 4A,B). Synthetic cargo localization in the presence of TRAK1 or TRAK2 was used to define peripheral and centrosomal regions, respectively, in these polarized cells (Fig. 4B,C) (45). Similar to synthetic cargo, TRAK1 expression shifted the distribution of mitochondria towards the periphery, while TRAK2 expression caused centrosomal clustering (Fig. 4A,B, fig. S11B). In contrast, peroxisomes did not change their distribution upon TRAK1 or TRAK2 expression (fig. S11C).

We next investigated the effect of DRH-mediated regulation on mitochondrial distribution. Similar to synthetic cargo, for TRAK1, the DRH-opening mutant produced centrosomal instead of peripheral mitochondrial localization (Fig. 4D, fig. S11D) and maintained such centrosomal clustering for TRAK2 (Fig. 4E, fig. S11E). To investigate dynein-kinesin competition for mitochondrial transport, we used the DRH-Spindly combined mutant to decrease dynein binding. Compared with the DRH mutant alone, the DRH-Spindly mutant promoted kinesin over dynein transport of mitochondria for TRAK2, but not TRAK1 (Fig. 4D,E; fig. S11D,E). These results mirror differences in kinesin activation by DRH-Spindly mutants for TRAK1 and TRAK2 observed with synthetic cargo (Fig. 2H,I; fig. S7C).

To test whether JNK2-mediated signalling promotes centrosomal clustering of mitochondria in response to oxidative stress, we treated either control or JNK2 knockdown cells with arsenite. Arsenite caused mitochondria to be depleted from the periphery and accumulate in the centrosomal region in control cells (Fig. 4F), and this effect was attenuated in JNK2 knockdown cells (Fig. 4F). JNK2 knockdown alone did not significantly affect mitochondrial distribution (Fig. 4F, fig. S11F). Collectively, these data suggest that the mechanism for coordinating microtubule-based motor transport by the TRAK DRH uncovered using the synthetic cargo assay also governs mitochondrial distribution.

## Dynein regulation by related adaptor proteins

We next tested whether the DRH regulatory switch mechanism uncovered in vertebrate TRAKs is present in other dynein adaptors. Milton, the Drosophila TRAK homolog, lacks the vertebrate-conserved CC1-box residues (A129, A130) and one of the two dynein LIC-mimicking DRH residues (F95/M95) (fig. S12A). Consistent with a lack of LIC binding, Milton solely transported dMiro cargo to the periphery when expressed in RPE-1 cells (fig. S12B), suggesting that it primarily mediates kinesin transport.

We also examined JIP3 and JIP4, lysosome adaptor proteins that bind both dynein and kinesin (46–49), and have a putative autoinhibitory helix (46) (fig. S13A). Using our synthetic cargo assay, we found that mutating autoinhibitory residues that prevent dynein LIC binding in JIP3 (fig. S13B) and analogous residues in JIP4 (Fig. 4G, fig. S13C) led to perinuclear accumulation, indicative of dynein activation. The autoinhibitory helices of JIP3 and JIP4 are both followed by a conserved stretch of serine residues (fig. S13A) that have been reported to be phosphorylated (50). Mutating these serines to phosphomimetic glutamate residues strongly activated dynein transport by JIP4, but not JIP3 (Fig. 4H; fig. S13D). These results suggest that JIP4 dynein transport, similar to TRAK2, could be regulated by a kinase that phosphorylates residues adjacent to an autoinhibitory helix.

## Conclusions

Using synthetic cargo and native mitochondria, we found that dynein and kinesin transport is coordinated by the conformational state of the TRAK dynein regulatory helix (DRH). Our data suggest a model in which anterograde transport occurs when the TRAK DRH is closed, preventing dynein LIC binding, and allowing kinesin to bind the TRAK coiled-coil (Fig. 4I). This anterograde TRAK conformation remains associated with dynein-dynactin through its Spindly motif, allowing kinesin to carry dynein-dynactin as an inactive cargo, as previously observed in vitro (25). We hypothesize that additional interactions between TRAK1 DRH and the downstream coiled-coil region facilitate TRAK1’s higher affinity for kinesin compared to TRAK2. In the retrograde TRAK conformation, the DRH is open, allowing the dynein LIC to engage with the TRAK CC1-box and aligning the dynein motor domains in parallel for processive movement (51). Our results also suggest that this active dynein conformation displaces kinesin from TRAK, mitigating interference from the opposing motor.

Our results also raise questions about the roles and regulation of TRAK1 and TRAK2 in mitochondrial transport. TRAK2, which we demonstrated activates dynein transport in response to stress through MAP kinase phosphorylation adjacent to the DRH, is enriched in the brain (11), which is particularly sensitive to ROS generation (52). In our cell culture model, TRAK1 promoted kinesin transport only. However, TRAK1 contains a fully functional CC1-box that can activate dynein transport through DRH displacement, and thus may respond to another physiological signal. Beyond the stress-activated DRH switch described here, there are likely additional signalling mechanisms involving metabolic sensing (28, 31), and other regions of TRAK (13, 16). Thus, the TRAKs, and other motor adaptors, may integrate multiple signals regarding cell state and translate such information into motor activation and organelle redistribution.

## Supplementary Material

Movie_S1

Movie_S2

Gladkova_Supplemental_Material

## Data Availability

All data are available in the main text or the supplementary materials. All reagents and materials are available from C.G. (gladkoc@mskcc.org) upon reasonable request. All image analysis code is available at Zenodo: Synthetic cargo accumulation (36), Synthetic cargo tracking (39), and Micropatterned cell analysis (45).
