# Supplementary material for: A molecular switch for coordinating kinesin and dynein transport of mitochondrial cargo
Source: Science. Author manuscript; Available in PMC 2026 Sep 5. (PMC13545989; doi:10.1126/science.aeh1475)
Supplement: Supplementary file 1 [file NIHMS2205143-supplement-Gladkova_Supplemental_Material.pdf]

Science
AAAS

Supplementary Materials for

A molecular switch for coordinating kinesin and dynein transport of mitochondrial cargo

Christina Gladkova, Maria G. Paez-Segala, William P. Grant, Mark Kittisopikul, Samuel A. Myers, Yuxiao Wang, Ronald D. Vale

Corresponding author: gladkoc@mskcc.org or valer@wi.mit.edu

The PDF file includes:

Materials and Methods
Figs. S1 to S14
References (*53-61*)

Other Supplementary Materials for this manuscript include the following:

Movies S1 and S2

Materials and Methods

Molecular Biology

Synthetic cargo constructs (Miro1-sfGFP-FLAG-I3-01 (Addgene ID: 248233), Miro1-Halo-FLAG-I3-01 (Addgene ID: 248236), dMiro-sfGFP-FLAG-I3-01 (Addgene ID: 248275), FRB-sfGFP-FLAG-GB1-I3-01 (Addgene ID: 248277)), TRAK-mScarlet fusion constructs (Addgene ID: 248234 (TRAK1 1-702), 248267 (TRAK1 fl), 248235 (TRAK2 1-700), 248268 (TRAK2 fl)), TRAK-mScarlet-FKBP fusion constructs (Addgene ID: 248253 (TRAK1 1-702), 248254 (TRAK2 1-700)), truncated Milton-mScarlet fusion constructs (Addgene ID: 248276 (Milton 1-756)) and truncated JIP-mScarlet-FKBP fusion constructs (JIP3 1-606, JIP4 1-583) were assembled using the NEBuilder HiFi DNA Assembly Master Mix (New England Biolabs) into a 2nd generation lentiviral transfer vector containing a SFFV promoter to drive expression as well as cPPT and WPRE elements to enhance transduction and expression respectively (gift from Kara L. McKinley).

Miro1, dMiro, Milton, TRAK1, TRAK2, JIP3 and JIP4 sequences were obtained as cDNA, gBlocks from IDT, or cloning vectors (Twist). I3-01 (*35*), sfGFP, GB1, mScarlet, Halo, FLAG, FRB and FKBP sequences were amplified from laboratory vector stocks. A monomerizing V206R mutation was introduced in the sfGFP sequence. Synthetic cargo Miro1 and dMiro fusion plasmids contain Miro1 truncated before the transmembrane domain.

Mutagenesis was performed by the amplification of two fragments overlapping at the mutation site using primers designed with the QuickChange Primer Design Program (Agilent) along with facing vector-homologous primers, and their assembly back into the original vector using NEBuilder HiFi DNA Assembly Master Mix (New England Biolabs). Generated TRAK variants are available on Addgene: TRAK-mScarlet fusions (Addgene ID: 248237 – 248252), full length TRAK-mScarlet fusions (248269 - 248274) and TRAK-mScarlet-FKBP fusions (248255 – 248266).

Cell culture, cell line generation and perturbation

hTERT RPE-1 (cTT33.1 carrying inducible Cas9, gift from Kara L. McKinley and Iain Cheeseman) and Lenti-X HEK293T cells for lentiviral packaging (Takara Bio) were cultured in complete media: high-glucose DMEM (Gibco) supplemented with 10% tetracycline negative FBS (Corning) and 1x Penicillin-Streptomycin-L-Glutamine (Corning). For passage, cells were dissociated using 0.05% Trypsin/0.53mM EDTA (Corning) following a PBS rinse. Low cell passage number was maintained, and cells were checked regularly for Mycoplasma using the MycoAlert PLUS Mycoplasma Detection kit (Lonza).

Stable cell line generation

Stable cell lines were generated by lentiviral transduction of the parent hTERT RPE-1 clone, and the transduced cell population was further enriched by FACS. A polyclonal population was used for all experiments, all comparisons are drawn between cells handled (transduced, gated and grown) identically. Briefly, lentivirus was generated by transfecting 1x10^6 Lenti-X HEK293T cells with transfer vector (2 µg), packaging vector (1 µg) and VSV-G envelope vector (0.5 µg) using Lipofectamine LTX reagent (Thermo Fisher) and Opti-MEM I Reduced Serum Medium (Gibco). Transfection-mix containing media was exchanged the following day, and crude viral supernatant was harvested 72 hours after transfection. Obtained viral supernatant for Miro1-FP-FLAG-I3-01 and TRAK-mScarlet fusions (20 µL) was mixed

with hTERT RPE-1 cells (0.05x10^6) in suspension containing polybrene at 5 µg/mL. The resulting mixture was centrifuged in a culture plate at 1000xg for 1h in 24-well tissue culture dishes (estimated MOI 100). To generate Miro1 synthetic cargo cell lines, cells were expanded for FACS once confluent. Gates were set based on untransduced or hTERT RPE-1 cells transduced with virus containing a single FP to select for low/medium expressors. Post-hoc computational analysis of the combined synthetic cargo dataset revealed relatively low (< 5 fold) variation of TRAK expression level across all variants. TRAK expression level differences were uncorrelated with synthetic cargo localization phenotype. To generate dMiro and FRB synthetic cargo cell lines, cells were first infected with the corresponding synthetic cargo construct and FACS sorted to isolate a polyclonal cargo-expressing cell population. Subsequently, cells were transduced with lentivirus encoding TRAK- or JIP-mScarlet-FKBP fusions (FRB cargo) or Milton mScarlet fusions (dMiro cargo), expanded and imaged without further FACS sorting (median transduction efficiency ~65%). To express TRAK variants for the micropatterning assay cells were transduced with lentivirus encoding full length TRAK mScarlet fusions, expanded and plated on micropatterned 96 well plates.

Gene knockdown using siRNA

SilencerSelect (JNK1 - s11152, JNK2 - s229708, JNK3 - s11161, p38δ - s11164), Validated SilencerSelect (KIF5B - s731, DYNC1H1 - s4202) and the SilencerSelect Negative control #1 siRNAs were ordered from Thermo Fisher. siRNAs were reverse transfected at a final concentration of 25nM using Lipofectamine RNAiMAX (Thermo Fisher) and Opti-MEM I Reduced Serum Medium (Gibco). For imaging, 0.5x10^4 hTERT RPE-1 cells were seeded into 96 square-well glass-bottom imaging dishes (Matriplate MGB096-1-2-LG-L). For WCL analysis, 2.5x10^4 hTERT RPE-1 cells were seeded into 12-well tissue culture dishes. For immunoprecipitation, 2.5*10^6 were seeded into 15 cm culture dishes. Cells were imaged, fixed, harvested or re-plated on micropatterned 96 well plates 72 hours after siRNA transfection.

Drug treatments

As above, 96- and 12-well dishes were used for imaging and WCL analysis respectively. Drug concentration and treatment duration is indicated in the figures and legends where applicable. A 2x solution of nocodazole or arsenite was prepared in complete culture media, which was applied at a 1:1 ratio with pre-existing culture media. Mock treatment consisted of addition of media alone. For imaging, cells were either fixed at the treatment endpoint (arsenite) or imaged live at the treatment endpoint (nocodazole). For whole cell lysate immunoblot analysis, cells were harvested at the treatment endpoint.

Image acquisition – synthetic cargo accumulation

Sample preparation (Miro1 and dMiro cargo)

Cells were seeded into 96 square-well glass-bottom imaging dishes (Matriplate MGB096-1-2-LG-L), such that they reach a density of ~4x10^4 cells/well at the time of imaging, and at least a day prior to imaging. For assessing the effects of TRAK variants / Milton, cells were stained with Hoechst 33342 (Thermo Fisher) prior to live imaging. For most cell perturbation experiments, cells were fixed using 4% PFA (Electron Microscopy Sciences) diluted in PHEM buffer (60 mM PIPES (from pH = 6.8 stock), 25 mM HEPES (from pH = 7.4 stock), 10 mM EGTA, 5 mM MgCl2), rinsed with PBS and stained with Hoechst 33342 prior to imaging.

Sample preparation (FRB cargo)

Cells were seeded into 96 square-well glass-bottom imaging dishes (Matriplate MGB096-1-2-LG-L), such that they reach a density of ~4x10^4 cells/well at the time of imaging, and at least a day prior to imaging. Cells were stained with Hoechst 33342 (Thermo Fisher) prior to live imaging. Cells were imaged as described below using the live high content JOBS acquisition at 3h intervals. After the first timepoint was collected, rapamycin was added at a final concentration of 0.5 µM. To ensure saturating dimerization and transport-dependent localization, FRB cargo accumulation was analysed as described below at the third timepoint, approximately 6h after rapamycin addition.

Image acquisition

All images were acquired on an Eclipse Ti2 inverted microscope (Nikon) equipped with a CSU-W1 spinning disk confocal unit (Yokogawa) using a 50 µm-pinholed disk, and controlled via NIS-Elements (Nikon, AR 5.42.03). The system was equipped with Hamamatsu ORCA-Fusion BT sCMOS cameras (Hamamatsu) and operated in 'ultra-quiet' mode with an exposure time of 200 ms. The environment was maintained at 37°C with 5% CO2 using either a cage or a stage top incubation system (Okolab). Cells were excited with 405-nm (nuclei), 488-nm (synthetic cargo, Tom20 immunofluorescence), 561-nm (TRAK), and 640-nm (MitoTracker DeepRed, Tom20 immunofluorescence) laser lines and emission was collected using one of the following objectives (Nikon) and standard filter sets: 20x/0.75 NA Plan Apo λ or 20x/0.8 NA Plan Apo λ D (high content acquisition), 60x/1.4 NA Plan Apo λ D (Fig. 1C), 100x/1.45 NA Plan Apo λ D (fig. S3B, Video 1). For typical high content acquisition using the 20x objectives, the JOBS module was utilized and 16 µm z-stacks were acquired using a piezo-driven stage with a 2 µm step size (Mad City Labs).

Image acquisition - synthetic cargo particle tracking

Sample preparation

Cells were seeded into 96 well glass-bottom imaging dishes as above. Prior to imaging, cells were stained with 100 nM PA-JF646-Halo photoactivatable dye for 30 min in complete media (gift from L. Lavis, Janelia Research Campus33) and washed 3x for 10 min with complete media.

Image acquisition

Images were acquired on the same spinning disk confocal microscope described above using a 50 µm-pinholed disk operated in 'ultra-quiet' mode with an exposure time of 200 ms/frame. Prior to photoactivation, cells were either imaged using transmitted light (no TRAK expression) or the 561 nm laser (TRAK1 or TRAK2 expression) to infer the cellular footprint and nuclear position necessary to determine position of photoactivation and to enable computational track reorientation (see analysis below). For simultaneous imaging and dye activation, live acquisition was started before stimulating cells using a 405 nm laser controlled via an Opti-Microscan for photostimulation module targeting a circular ROI (1.5 µm diameter) halfway between the nucleus and cell periphery. Emission was collected using a 100x/1.45 NA Plan Apo λ objective (Nikon) using a 705/72 nm filter.

Image acquisition – mitochondria, peroxisome and synthetic cargo in micropatterned cells

96 well plate micropatterning

The PRIMO system (Alveole) was used to pattern crossbow micropatterns on 96 square-well glass-bottom imaging dishes (Matriplate MGB096-1-2-LG-L). Plate cleaning: plates were stirred in a 5% solution of Hellmanex™ III (Sigma-Aldrich) overnight at 50°C, rinsed 10x for 10 min in warm water, blow dried, and subjected to plasma cleaning for 2 min. Wells were immediately covered with 120 µl of Poly-L-Lysine (PLL, Sigma-Aldrich, P4707) and incubated O/N at room temperature. PLL was washed 3x with DI water, and the plate dried at 50°C. Once dry, a 100 mg/mL solution of 5K mPEG-SVA (Laysan Bio) was prepared in 0.1M HEPES (pH=8.5), and 100 µl immediately added to each well to passivate the surface. After a 1h incubation, the mPEG-SVA solution was removed, plate washed 4x with DI water and dried at 50°C. Immediately prior to patterning, a layer of photoactivatable gel was deposited in each well by the addition of 80 µl of a PLLP gel solution (20 µl PLLP gel (Alveole) and 1.4 µl surfactant (Alveole) per 1 mL of DI water) and subsequent drying at 50°C. UV patterned illumination of each well (1600 patterns, 40 mJ/mm^2) was carried out using the PRIMO device (Alveole) controlled by the HCS wizard available through the Leonardo plugin (Alveole). Following illumination, the plate was washed 3x with PBS, incubated with 70% EtOH for 2-10 min, and washed 4x with PBS. Finally, each well was coated with 80 µl of 8 µg/mL fibronectin HiLyte Fluor 647 (Cytoskeleton) diluted with PBS supplemented with 0.01% Pluronic F-127 (Thermo Fisher) for 45 minutes, and rinsed 3x with PBS.

Sample preparation

3000 cells were seeded in each well using conditioned media (1:1 mixture of fresh complete media and complete media collected from a confluent hTERT RPE-1 culture). One hour after seeding, conditioned media was exchanged for fresh media, or media containing 100 µM arsenite as indicated. One hour after media exchange, wells were fixed using 4% PFA (Electron Microscopy Sciences) diluted in PHEM buffer (60 mM PIPES (from pH = 6.8 stock), 25 mM HEPES (from pH = 7.4 stock), 10 mM EGTA, 5 mM MgCl2), permeabilized using 0.5% Triton-X in PHEM, blocked using 3% BSA in PBS and incubated overnight at 4°C with primary antibody (Tom20; abcam, ab186735 or PMP70; Sigma, SAB4200181). Subsequently, wells were washed and incubated with AlexaFluor-labelled secondary antibody (Thermo Fisher, A-11001 or A-11008) and Hoechst 33342.

Image acquisition

Images were acquired on the system described for synthetic cargo above without environmental control and using the 100x/1.45 NA Plan Apo λ D objective. First, a tile image (approx. 2x4 mm) was acquired to mark the positions of uniform and, TRAK-expressing cells where applicable. 12 µm z-stacks were acquired using a piezo-driven stage with a 0.2 µm step size (Mad City Labs) for selected positions. Acquired z-stacks were denoised using the Denoise.ai function of the NIS.ai package (Nikon) to remove shot noise. Images were visually inspected, and those fitting the following criteria were included in the analysis: stereotypical pattern, single cell per pattern, nucleus does not occupy the entire peripheral region, cell confined to the micropattern, cell fully populates micropattern, TRAK signal detected (where applicable), free of fluorescent debris.

Image analysis

Synthetic cargo accumulation

To quantify the accumulation of Miro1, FRB and dMiro synthetic cargo, a set of custom scripts were developed to fully automate the measurement of perinuclear and peripheral cargo accumulation in the whole image dataset: https://doi.org/10.5281/zenodo.20263176 (*36*). This was developed based on a Cell Profiler pipeline with the assistance of Claude Opus 4.5/Opus 4.6/Opus 4.7 (Anthropic).

First, a Cell Profiler (4.2.4) pipeline was developed for analysis of high content imaging of synthetic cargo accumulation (*53*), (fig. S2). Nuclei (30-100 px diameter) were segmented from a median filtered (3px) hoechst channel using 3-class otsu thresholding. The threshold correction factor was adjusted (0.35 to 0.92) for best thresholding performance for each biological replicate. Images with bright debris in the nuclear channel were excluded based on the variance of the calculated threshold value (25/2278 images).

To measure perinuclear accumulation of the synthetic cargo, the segmented nuclear mask was expanded by 10 px to define the perinuclear region. A custom plugin was developed to measure the degree of pixel intensity inequality within the perinuclear region mask of the synthetic cargo image by calculating the Gini coefficient (https://doi.org/10.5281/zenodo.13150979). The Gini coefficient takes values from 0-1, and compares the observed pixel frequency distribution to a hypothetical case where all pixels have a uniform intensity value. To identify edge spots, the segmented nuclear mask was expanded by 15 px and subtracted from the synthetic cargo image such that only pixels corresponding to peripheral cell areas remained. Peripheral signal accumulation was detected using global robust background thresholding in the resulting masked image (threshold= 2× (robust median + 2σ), 3-80 px diameter). The intensity of synthetic cargo signal for all edge spots in a single image was recorded along with total synthetic cargo signal intensity.

In addition to replicating steps in the pipeline described above, the custom python scripts handle downstream data processing. To generate a per-image perinuclear accumulation score, the median Gini coefficient for all cells in a given image was calculated. To distinguish true peripheral accumulation spots from perinuclear accumulation that escaped the perinuclear masking step, we excluded all spots within 3px of the perinuclear mask. To generate a per-image peripheral accumulation score, the sum intensity for all accepted spots was normalized to the total unmasked synthetic cargo intensity. These per-image values were normalized to a control condition in each biological replicate measurement and plotted and treated as a single technical replicate.

Because FRB cargo images contain a mixture of TRAK- or JIP- mScarlet fusion expressing cells and untransduced cells (median transduction efficiency was ~65%), we introduced a filtering step to exclude untransduced cells from the per-cell Gini coefficient analysis. Cells were considered mScarlet-positive if their mean 561 intensity within the perinuclear region was at or above the 90th percentile of the mean intensity observed in the untransduced control population imaged on the same plate.

Synthetic cargo particle tracking

Acquired time-lapse series were denoised using the Denoise.ai function of the NIS.ai package (Nikon) to remove shot noise. Particle trajectories were generated using the TrackMate

Fiji plugin (*54*). To reduce linking artifacts caused by high particle density immediately following photoactivation, only frames acquired 40-120s (200ms per frame, 400 frames) after photoactivation were included in the analysis. A Laplacian of Gaussian detector was used to identify synthetic cargo particles with an estimated object diameter of 0.4 µm, employing sub-pixel localization. The linear assignment problem tracker was used to generate trajectories with a max linking distance and a gap closing distance set to 1.0 µm. Only tracks containing 6 or more spots were included in the analysis to reduce the effect of out-of-plane diffusion. For each cell, coordinates of the bleach spot and the approximate nucleus centroid were recorded from a bright-field or TRAK channel image and used to generate a cell orientation vector between the nucleus to the bleach spot (periphery). Kymographs in Fig. 1E were generated in ImageJ by re-slicing acquired image stacks using a rectangular ROI (8x0.65 µm) as indicated in fig. S3B.

Trajectories acquired for cells expressing no TRAK (14 cells, 3,789), TRAK1 (21 cells, 24,392), TRAK2 (47 cells, 32,669) across three different days were exported from TrackMate and analysed using a custom script: https://doi.org/10.5281/zenodo.20218242 (*39*). Briefly, all tracks were origin aligned and reoriented according to the experimentally determined cell orientation vector, such that the cell periphery is in the +ve y direction and the nucleus in the -ve y direction. By comparing the properties of particle tracks obtained with and without TRAK expression, we defined tracks with >3.0 µm maximum distance travelled as processive (no TRAK: 8, TRAK1: 573, TRAK2: 270). To assess the distribution of track properties, the final position of origin-aligned reoriented tracks, the mean instantaneous speed per track and the mean instantaneous speed per track per final position was plotted for all processive tracks in the TRAK1- and TRAK2-expressing condition.

Mitochondria, peroxisomes and synthetic cargo in micropatterned cells

To quantify mitochondria, peroxisome and synthetic cargo signal distribution in micropatterned cells, a set of custom scripts were developed: https://doi.org/10.5281/zenodo.20269805 (*45*).

Images were loaded using the nd2 library (v0.10.3) with xarray (v2025.9.0) for labelled multidimensional arrays. Analysis pipelines were prototyped and visualized interactively using Marimo (v0.17.8), a reactive Python notebook environment. All analysis was performed in Python 3.12 using packages managed via the Pixi environment manager.

A micropattern template was defined as an SVG file and rasterized to 1326 × 1326 pixels using CairoSVG (v2.8.2) and PyMuPDF (v1.26.4). The template was zero-padded to 2048 × 2048 pixels for frequency-domain matching. The micropattern center was localized in each image by normalized cross-correlation in frequency space using 2D FFTs (NumPy v2.3.3). The 640 nm channel z-sum projection was used as the reference signal. A template matching score (fractional overlap with Otsu-thresholded image) was computed for quality control.

A 1024 × 1024 pixel region centered on the matched template was extracted for each fluorescence channel and saved as NetCDF4 (v1.7.3) files. The 405 nm (Hoechst) and 640 nm (template) channels were stored as z-sum projections, while the 488 nm (mitochondria, peroxisomes, synthetic cargo) channel was stored as a maximum-intensity projection to reduce noise. Background was estimated from the empty leftmost and rightmost 128-pixel column strips and subtracted. Experimental conditions and well assignments across plates were specified in a structured Excel spreadsheet read using fastexcel (v0.18.0) and Polars (v1.36.1).

For quantification, the MaxIP was rescaled to a normalized intensity (0.1-99.9 percentile) before bg subtraction. To quantify the directional distribution of mitochondrial signal, a fixed

polygonal wedge was defined in the cropped frame with its apex at the bottom extreme of the micropattern and bounded by the upper tangent points of the arch. For each cell, radial distance from the apex was computed and binned into sixty 1 µm bins. The intensity within each radial bin and within the wedge was summed and expressed as a percentage of total. Two regions were defined based on the TRAK1-mediated kinesin and TRAK2-mediated dynein synthetic cargo localization: peripheral (41-56 µm) and centrosomal (18-33 µm). Per-cell mitochondrial fractions in these regions were compiled into CSV files using Polars (v1.36.1).

To display 488 nm channel distribution for all cells within each condition, unscaled background-subtracted MaxIP images were spatially aligned by center-padding to a common bounding box and summed, then divided by the number of input cells to produce a mean projection image per condition. Mean projections were saved as 16-bit TIFF files using scikit-image (v0.25.2).

Biochemistry

Immunoprecipitation

For each replicate, cells from a confluent 15-cm dish were washed with, and scraped into ice-cold PBS supplemented with cOmplete Protease Inhibitor Cocktail, PhosSTOP (Roche) and 1 µM Thiamet G (Tocris), centrifuged at 200xg, and frozen at -80°C. FLAG immunoprecipitation protocol was based on the resin manufacturers recommendation (ChromoTek DYKDDDDK Fab-Trap™ Agarose, Proteintech). Cells were lysed in IP buffer (25 mM HEPES (from pH = 7.4 stock), 50 mM KOAc, 2 mM MgOAc, 1 mM EGTA, 1% v/v glycerol, 1x cOmplete Protease Inhibitor Cocktail (Roche), 1x PhosSTOP (Roche), 1 µM Thiamet G (Tocris), 0.5 mM TCEP (Sigma-Aldrich), 0.5 mM MgATP (Thermo Fisher), 1 mM PMSF (Calbiochem)) supplemented with 0.2% Triton X-100. Lysate was clarified by centrifugation at 17,000xg and diluted 2:3 with IP buffer. Diluted lysate was applied to DYKDDDDK Fab-Trap Agarose resin equilibrated with IP buffer, resin washed 3x with IP buffer, and protein eluted directly in SDS-sample buffer by boiling resin for 5 min at 95°C.

WCL preparation

Cells grown on a 12-well tissue culture dish were washed with ice-cold PBS. Cells were scraped into RIPA lysis buffer (Thermo Fisher) supplemented with 1x cOmplete Protease Inhibitor Cocktail (Roche), 1x PhosSTOP (Roche), 1 µM Thiamet G (Tocris), 0.5 mM TCEP (Sigma-Aldrich), 0.5 mM MgATP (Thermo Fisher), 1 mM PMSF (Calbiochem). Lysates were sonicated using 20% power (Q125 Sonicator, QSonic) for a total of 20s (5s on, 5s off). Total protein concentration was measured using a BCA Protein Assay (Thermo Fisher) and equalized across all samples analyzed in each experiment.

Immunoblotting

Immunoprecipitated or whole cell lysate samples were resolved on 4-12% Bis-Tris SDS NuPAGE gradient gels (Invitrogen) together with Broad Protein Standard (New England Biolabs), and transferred to a nitrocellulose membrane using the Trans-Blot Turbo Transfer system (Mixed MW transfer program, BioRad). For resolving the dynein heavy chain (DHC1, 532 kDa), 3-8% Tris-Acetate SDS NuPAGE gradient gels (Invitrogen) were used in combination with the High MW transfer program instead. Membranes were blocked in a 5% (w/v) milk solution (Fisher Sci) in PBS-T (PBS + 0.1% (v/v) Tween-20) and incubated overnight at 4°C

with primary antibody diluted in PBS-T + 3% (w/v) BSA + 0.02% (w/v) NaAzide (see below for a list of primary antibodies used). The membranes were then washed with PBS-T and incubated overnight at 4°C with secondary antibody diluted in a 5% (w/v) milk solution in PBS-T (HRP-conjugated Goat anti-Mouse or Goat anti-Rabbit Secondary Antibody, Thermo Fisher), washed with PBS-T and visualized using the SuperSignal West Pico PLUS Chemiluminescent Substrate and the ChemiDocTouch Imaging System (BioRad).

Uncropped membrane images and annotated input lanes are included in fig. S14. For quantification of immunoprecipitation experiments, immunoblot band intensity from signal corresponding to the GFP-labelled synthetic cargo and motor protein components of interest as indicated (KIF5B, DHC1, p150) was measured using ImageJ. The band intensity corresponding to co-eluting motor protein components was normalized by the intensity of the immunoprecipitated synthetic cargo (GFP band), and subsequently normalized with respect to a chosen condition as indicated to allow comparison between biological replicates.

The following primary antibodies were used: GFP (CST, 2555S), RFP (Proteintech, 6g6), KIF5B (abcam, ab167429), DHC1 (Santa Cruz Biotechnology, sc-514579), p150 (BD Biosciences, 610473), GAPDH (CST, 2118S), DIC (Sigma-Aldrich, MAB1618), p50 (BD Biosciences, 611002), SAPK/JNK (CST, 9252S), Phospho-SAPK/JNK (Thr183/Tyr185) (CST, 9251S), p38 MAPK (CST, 9212S), Phospho-p38 MAPK (Thr180/Tyr182) (CST, 9211S), O-GlcNAc [RL2] (abcam, ab2739), p38δ MAPK (CST, 2308T), TRAK1 (Sigma-Aldrich, HPA005853-100UL), OIP106/TRAK1 (Novus, NBP1-56761), TRAK2 (proteintech, 13770-1-AP), ALS2CR3/TRAK2 (Novus, NBP2-15370).

The p150 antibody (BD Biosciences, 610473) was further validated by gene knockdown using SilencerSelect DCTN1 (s3973, s3974) and the SilencerSelect Negative control #1 siRNAs from Thermo Fisher. The intensity of the only band detected using the antibody at an apparent MW of 180kDa was diminished by p150 knockdown (fig. S14).

Mass Spectrometry

To obtain sufficient protein for band excision from a Coomassie-stained protein gel, the immunoprecipitation protocol described above was scaled up eight-fold. An aliquot of the eluted SDS sample was used for validation of the experiment by IP-immunoblot (fig. S9A), and the remainder of the sample was resolved on a 4-12% Bis-Tris SDS WedgeWell Bolt gradient gel (Invitrogen), stained with InstantBlue Protein Stain (Expedeon) and bands excised as indicated in fig. S9B.

Gel bands were digested with trypsin (Promega), and desalted as previously described (*55*). Data was acquired on a Thermo Orbitrap Eclipse with ETD. Peptides were separated on a 60 min effective gradient from 2% solvent B (80% acetonitrile, 0.1% formic acid in water) to 30% B. The MS1 scan was measured in the Orbitrap at 60,000 resolution with a 50 ms injection time for 40,000 charges. The scan range was 350-1800 m/z for 2-8 plus precursors. MS2 scans were performed after a 2.0 m/z isolation window by the quadrupole, filled to 50,000 charges in 105 ms, followed by HCD at a normalized collision energy of 28%. Product ions were read out in the Orbitrap at 15,000 resolution. If 204.0867 or 138.0545 m/z +/- 15 ppm was detected in the MS2 scan, the mass spectrometer refilled for up to 250 ms for 100,000 charges before performing EThcD at 25% supplemental activation. Three microscans were permitted and product ions were measured in the Orbitrap at 7,500 resolution. Data was searched using Byonic (Protein Metrics, Inc) for protein identifications. Only proteins identified in the sample were further searched for HexNAc (STN), common 3, phosphorylation (STY) common 2. Cys

carbamidomethylation was a fixed modification and protein N-term acetyl and Met oxidation were rare. All discussed site assignments were validated by hand.

AlphaFold2 prediction and TRAK sequence analysis

Structure predictions were performed using AlphaFold2-Multimer (*41*) implemented via ColabFold (*40*) running on the Colab platform (Google). Two copies of wild type or mutant TRAK1 (aa 1-396) and TRAK2 (aa 1-914) were used to generate N-terminal coiled-coil predictions shown in Fig. 2A (top), fig. S5A. Two copies of TRAK1 or TRAK2 (aa 1-360) were used together with two copies of KIF5B (aa 821-963) to generate kinesin complex predictions shown in Fig. 2A (bottom), fig. S5B. Structural figures were generated using PyMol (www.pymol.org). pLDDT scores generated by AlphaFold2-Multimer were mapped onto the structure using indicated LUTs (Pymol LUT implementation designed by Konstantin Korotkov). Sequence alignment with structure prediction mapping in Fig 3, fig. S1, S12, S13 was generated using ESPript (*56*).

Statistics and reproducibility

Statistical analysis and graphing were carried out using Prism (v 10.6.1). Superplots representing imaging data show means for each biological replicate superimposed on the calculated phenotypic scores for individual fields of view (synthetic cargo) or cells (native cargo) from samples handled in parallel (see fig. S2), (*57*). Sample number, the number of biological replicates and employed statistical tests are described in each figure legend. For all normalized imaging data nested ANOVA analyses or nested t-tests were used depending on the number of comparisons made (*58*). For all normalized IP-immunoblotting data, one sample t-tests were used to distinguish values from the control value (=1). Because some experiments were carried out in parallel, comparing variants/perturbations to the same control condition, the same control distributions appear in multiple figures. TRAK1 (Miro1 synthetic cargo): Fig 2C, 2E; fig. S6D. TRAK2 (Miro1 synthetic cargo): Fig. 2D, 2F; fig. S4F, S6E. TRAK1 (Miro1 synthetic cargo, ctrl siRNA): fig. S4B, S4D. TRAK2 (Miro1 synthetic cargo, ctrl siRNA): fig. S4A, S4C. TRAK1 and TRAK2 (FRB synthetic cargo): fig. S8A, S8B, S8C. JIP4 (FRB synthetic cargo): Fig. 4G,H. JIP3 (FRB synthetic cargo): fig. S13B,D. TRAK1 (mitochondria): Fig. 4C,D. TRAK2 (mitochondria): Fig. 4C,E. All representative micrographs were selected from matching cell lines handled in parallel.

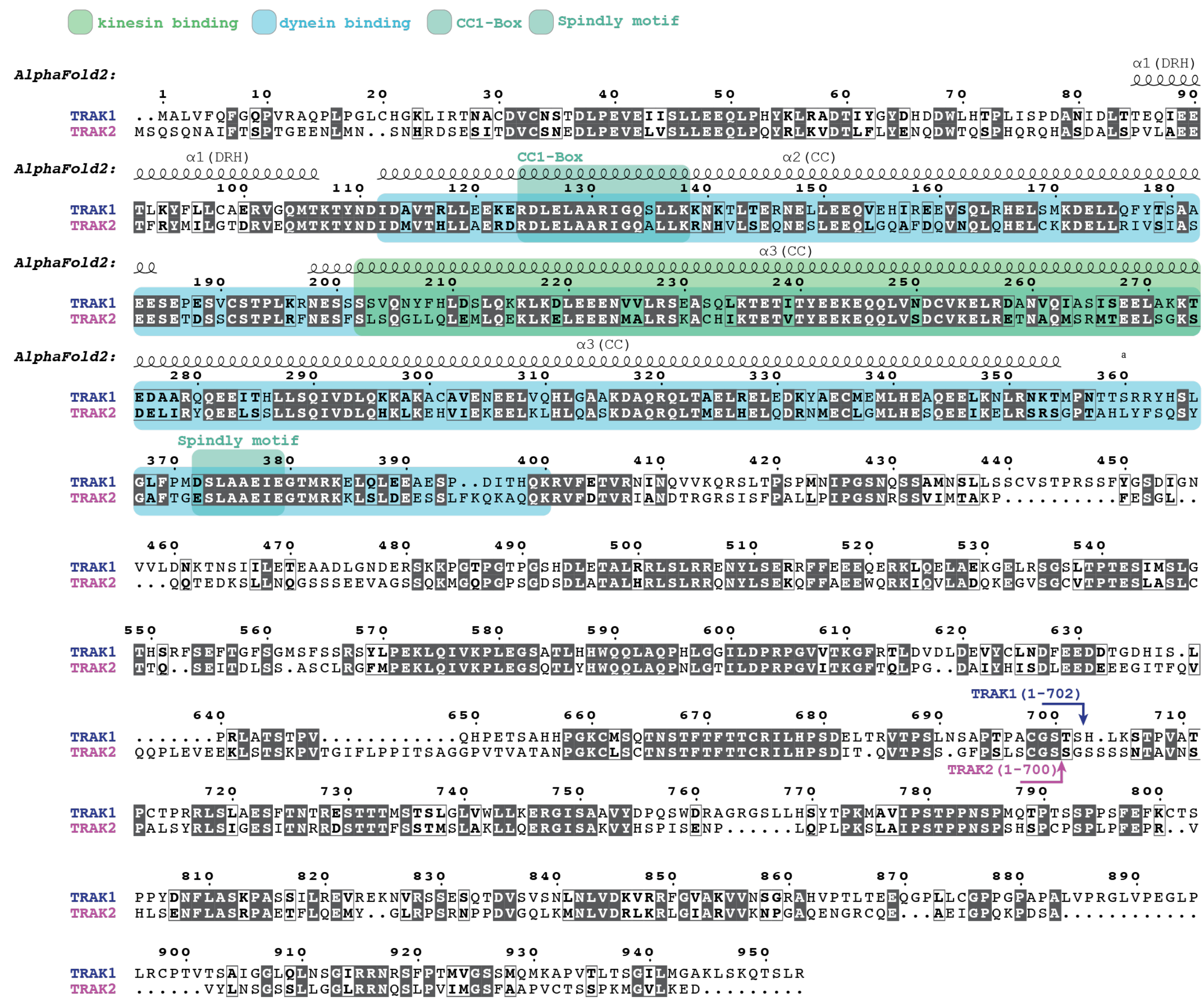

fig. S1. Sequence alignment of TRAK1 and TRAK2.

Sequence alignment of TRAK1 and TRAK2 with the following annotations: previously mapped binding regions for kinesin (green) and dynein (blue), CC1-Box and Spindly motif (cyan), secondary structure elements reliably identified by AlphaFold2 (see fig. S5), and C-terminal boundaries for the constructs used in this study.

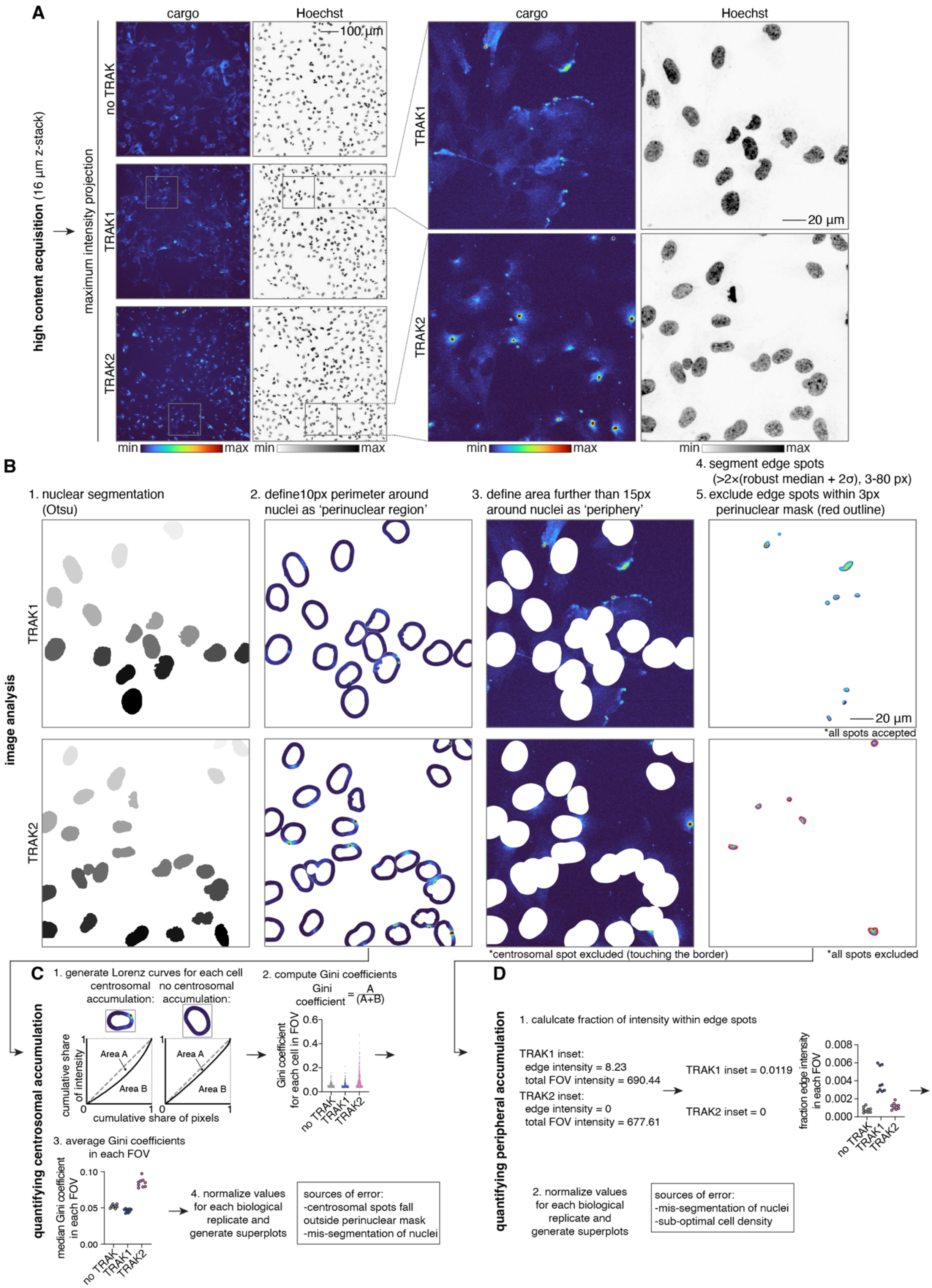
A
high content acquisition (16 µm z-stack)
maximum intensity projection
cargo
Hoechst
100 µm
no TRAK
TRAK1
TRAK2
20 µm
min
max
B
image analysis
1. nuclear segmentation (Otsu)
2. define10px perimeter around nuclei as 'perinuclear region'
3. define area further than 15px around nuclei as 'periphery'
4. segment edge spots (>2×(robust median + 2σ), 3-80 px)
5. exclude edge spots within 3px perinuclear mask (red outline)
*all spots accepted
*centrosomal spot excluded (touching the border)
*all spots excluded
C
quantifying centrosomal accumulation
1. generate Lorenz curves for each cell
centrosomal accumulation:
no centrosomal accumulation:
cumulative share of intensity
cumulative share of pixels
Area A
Area B
2. compute Gini coefficients
Gini coefficient = A / (A+B)
Gini coefficient for each cell in FOV
3. average Gini coefficients in each FOV
median Gini coefficient in each FOV
no TRAK
TRAK1
TRAK2
4. normalize values for each biological replicate and generate superplots
sources of error:
-centrosomal spots fall outside perinuclear mask
-mis-segmentation of nuclei
D
quantifying peripheral accumulation
1. calulcate fraction of intensity within edge spots
TRAK1 inset:
edge intensity = 8.23
total FOV intensity = 690.44
TRAK2 inset:
edge intensity = 0
total FOV intensity = 677.61
TRAK1 inset = 0.0119
TRAK2 inset = 0
fraction edge intensity in each FOV
2. normalize values for each biological replicate and generate superplots
sources of error:
-mis-segmentation of nuclei
-sub-optimal cell density

fig. S2. Quantifying synthetic cargo localization.

(**A**) Left: Maximum intensity projection images showing an entire field of view (FOV) acquired for cells expressing synthetic cargo (left) and labelled with Hoechst (right) at 20x magnification. Right: Insets for TRAK1 and TRAK2 conditions showing ~20 cells. (**B**) Example outputs from the Cell Profiler pipeline used for phenotype quantification for the inset shown in (A). Nuclear segmentation (left) was used to mask the synthetic cargo image and define a 'perinuclear region' for each cell (middle left) and the 'periphery' for each FOV (middle right). Peripheral accumulations of cargo were identified by a subsequent round of segmentation (right), and filtered based on their proximity to the perinuclear mask. A custom Python script replicating the steps illustrated was used to measure per-image centrosomal and peripheral accumulation of synthetic cargo. (**C**) Centrosomal accumulation was measured by quantifying the pixel intensity distribution in the perinuclear region of each segmented nucleus using the Gini coefficient. The Gini coefficient takes values from 0-1 and measures the inequality of pixel intensities in the perinuclear region by comparing the observed pixel frequency distribution to a hypothetical case where all pixels have a uniform intensity value (steps 1, 2). To generate a per-image centrosomal accumulation score, the median Gini coefficient for all cells in a given image was calculated (step 3). These per-image values were normalized to a control condition in each biological replicate measurement and plotted and statistically treated as a single biological replicate (step 4). (**D**) Per-image peripheral accumulation was measured by quantifying the fraction of synthetic cargo intensity within all accepted spots compared to the total unmasked synthetic cargo intensity (step 1). These per-image values were normalized to a control condition in each biological replicate measurement and plotted and statistically treated as a single biological replicate (step 2).

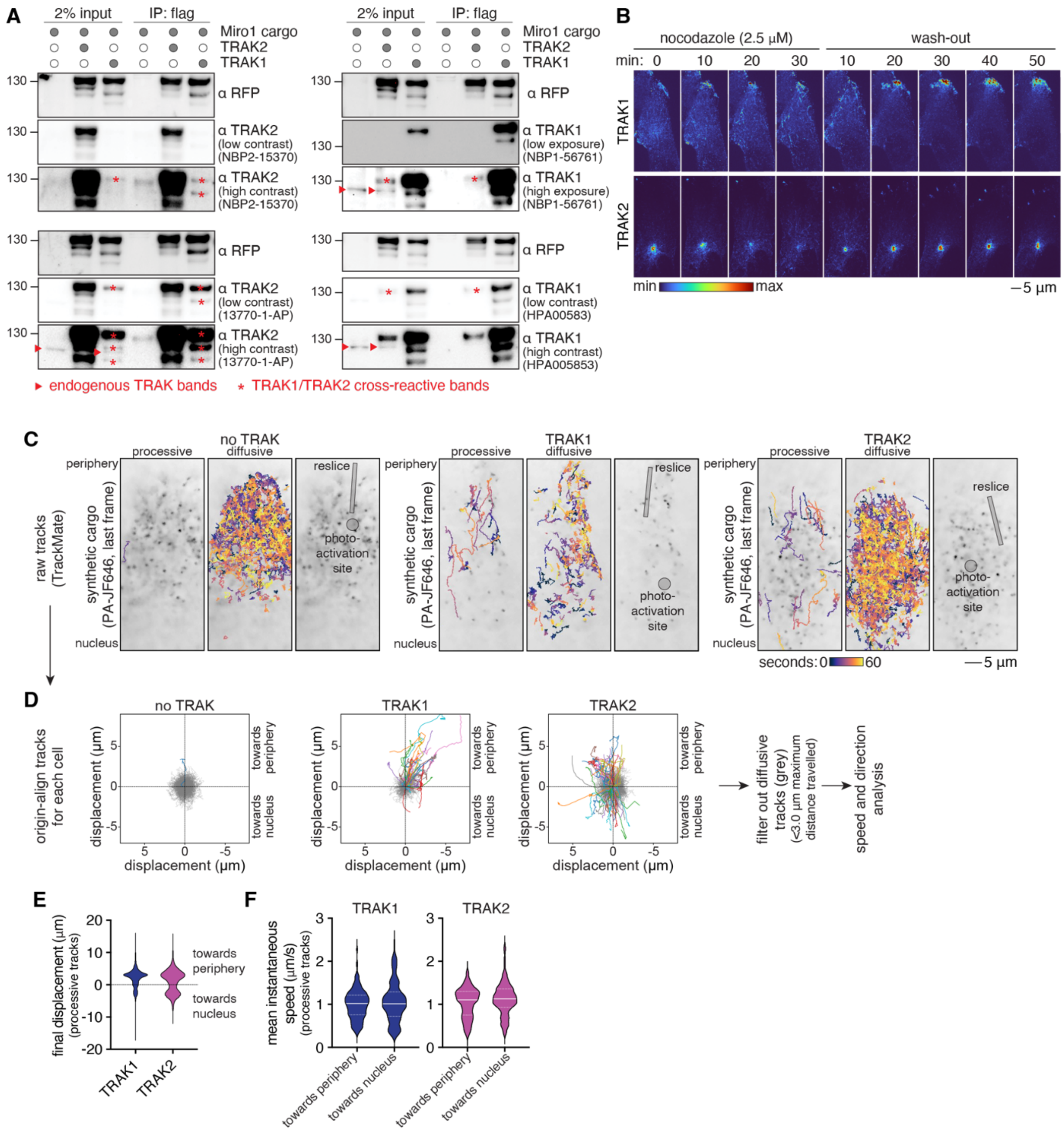

fig. S3. Synthetic mitochondrial cargo characterization.

(**A**) Immunoblot analysis examining the degree of endogenous TRAK association with FLAG-purified cargo from indicated cell lines. Two different antibodies against each TRAK were used (top or bottom). Synthetic cargo was immunoprecipitated from cell lines expressing cargo alone, or cargo along with TRAK1 or TRAK2 C-terminally truncated mScarlet fusions. TRAK antibody signal was compared with the RFP antibody signal to identify endogenous TRAK bands in the input lanes. These bands were not detected in the immunoprecipitated fractions, suggesting that endogenous TRAK does not associate with the Miro1-synthetic cargo, or cross-dimerize with overexpressed TRAK C-terminally truncated mScarlet fusions. Based on these results we estimate the abundance of overexpressed TRAK constructs to be approximately two

orders of magnitude higher than endogenous TRAK levels. (**B**) Maximum intensity projections of frames from time-lapse imaging of cargo localization following nocodazole treatment (2.5 µM, 30 min) and wash-out (see Movie S1). (**C**) Final frames from time-lapse imaging of photoactivated synthetic cargo of cells expressing no TRAK, TRAK1, or TRAK2 (see Movie S2). Overlaid are processive (left panel, >3.0 µm maximum distance travelled) or diffusive (middle panel, <3.0 µm maximum distance travelled) trajectories generated by TrackMate for the previous 60s. Photoactivation site and source area for kymographs in Fig. 1E are overlaid on the right panel. (**D**) Origin-aligned tracks from cells as in (C) that have been oriented such that the cell periphery is in the positive y direction and the nucleus in the negative y direction. Processive tracks are highlighted in colour, diffusive tracks are grey. (**E**) The distribution of final particle positions from origin-aligned and reoriented processive tracks as in (D) from the TRAK1 (n=21 cells, 573 tracks) and TRAK2 (n=47 cells, 270 tracks) datasets. (**F**) Mean instantaneous speed averaged over the length of processive tracks (maximum distance travelled >3.0 µm) from the TRAK1 and TRAK2 datasets as above, separated by direction of movement. This speed was 1.00 ± 0.02 µm/s for TRAK1 trajectories oriented towards the periphery (n=460 tracks), 1.06 ± 0.04 µm/s for TRAK1 trajectories oriented towards the nucleus (n=113 tracks), 1.04 ± 0.03 µm/s for TRAK2 trajectories oriented towards the periphery (n=172 tracks), 1.13 ± 0.04 µm/s for TRAK2 trajectories oriented towards the nucleus (n=98 tracks).

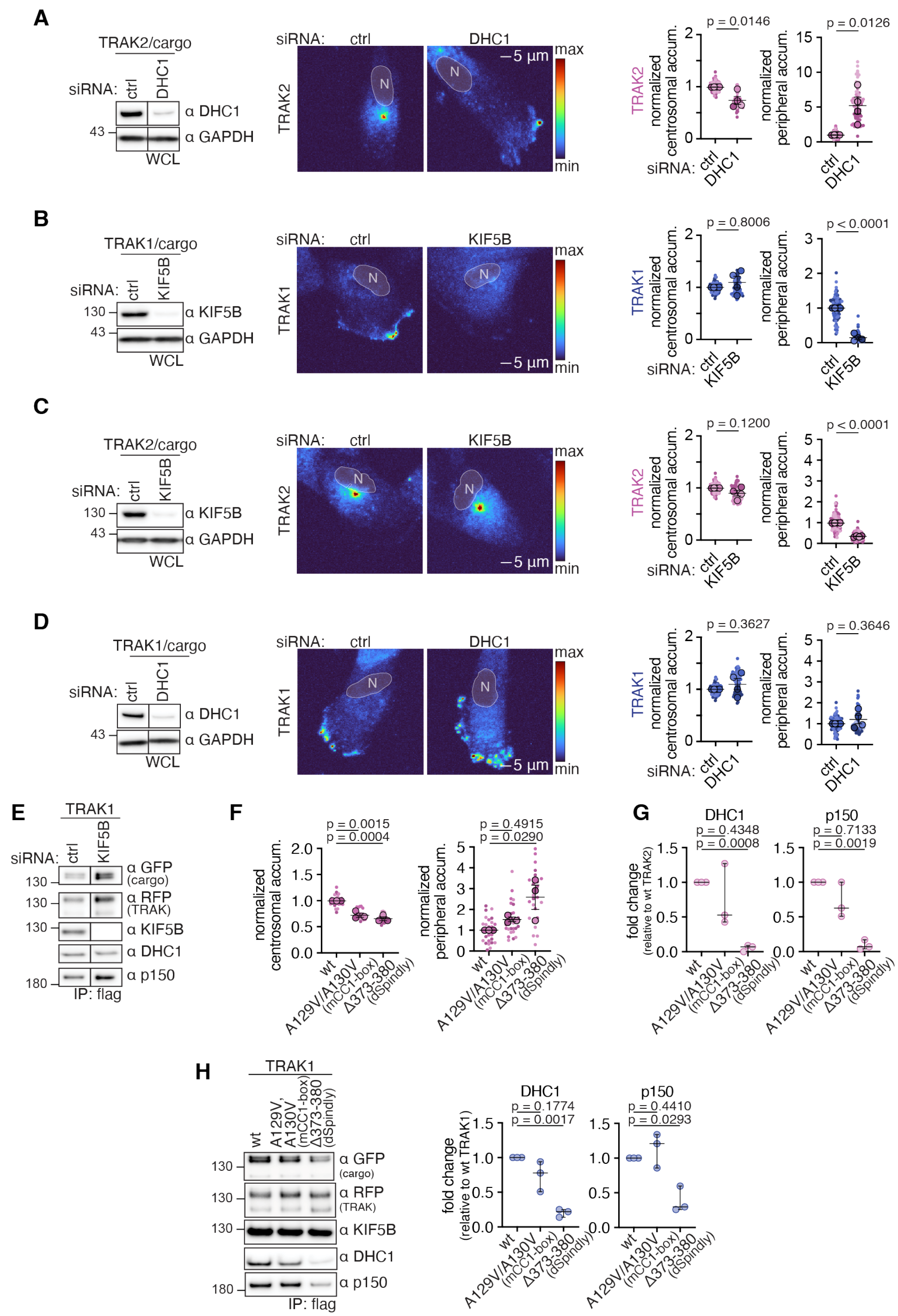

fig. S4. Motor-dependence of TRAK-mediated synthetic cargo transport.
(**A**), (**B**), (**C**), (**D**) Left: Immunoblot analysis of whole cell lysate from indicated cell lines showing the efficacy of DHC1 or KIF5B siRNA treatments compared to control. Middle and right: Representative maximum intensity projection images and quantification of cargo accumulation from indicated cell lines and siRNA treatments. Nested t-test (n=4), p-val ues indicated in the figure. Error bars show the mean and SEM. (**E**) Immunoblot analysis of the motor-adaptor components associated with FLAG-purified Miro1 cargo from TRAK1-

expressing cell lines treated with control or KIF5B siRNA. (**F**) Quantification of cargo accumulation from indicated conditions (A129V/A130V = CC1-box mutant, Δ373-380 = Spindly motif mutant). Nested ANOVA with Dunnett's test (n=3), p-values indicated in the figure. Error bars show the mean and SEM. Representative maximum intensity projection images shown in Fig. 1H. (**G**) Quantification of relative motor protein levels normalized to wt. One-sample t-tests (n=3), p-values indicated in the figure. Error bars show the median and 95% confidence interval. See Fig. 1I for representative immunoblot. (**H**) Left: Immunoblot analysis of the motor-adaptor components associated with FLAG-purified cargo from indicated cell lines. Right: quantification of relative motor protein levels normalized to wt. One-sample t-tests (n=3), p-values indicated in the figure. Error bars show the median and 95% confidence interval.

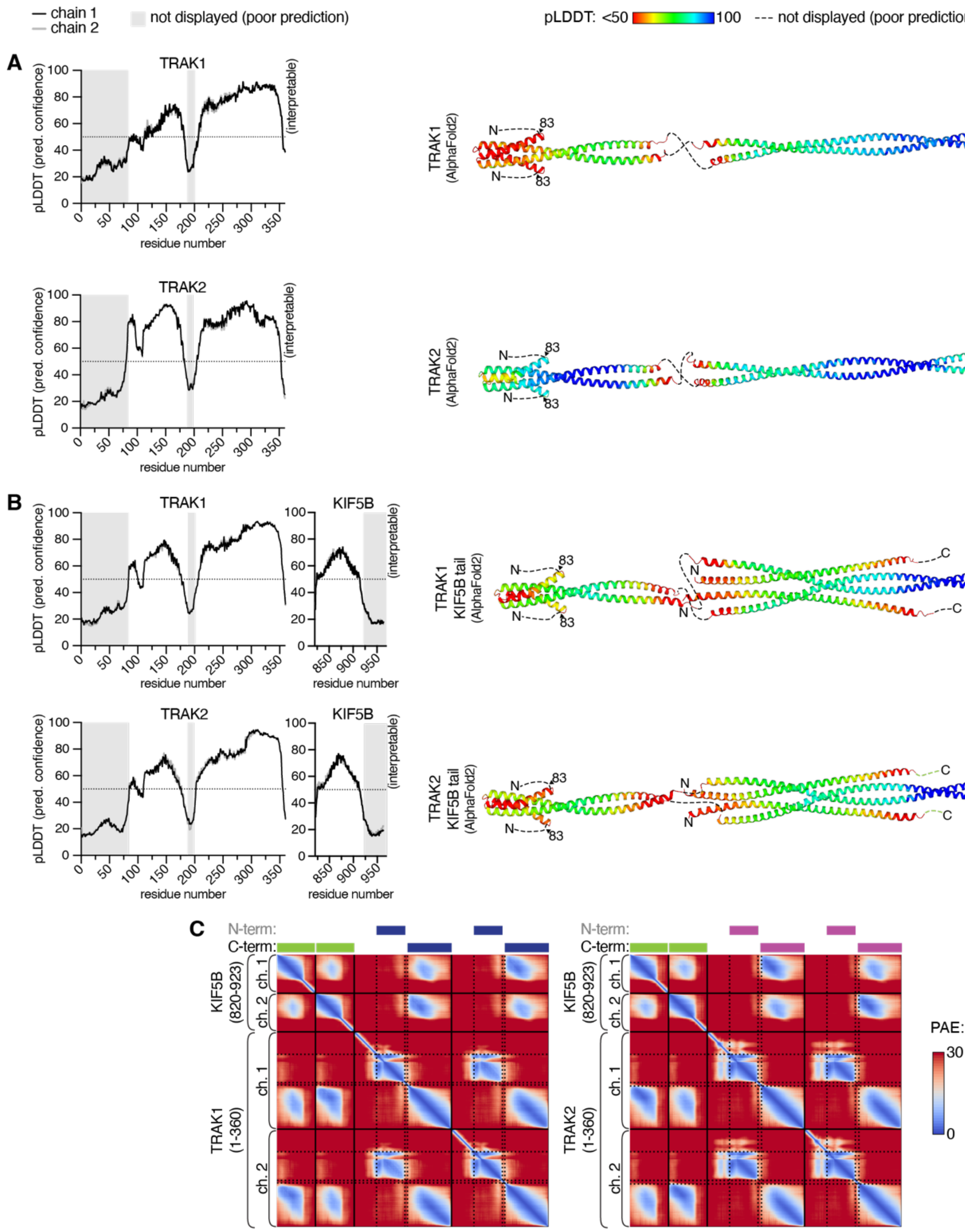

fig. S5. AlphaFold2 prediction confidence.

(**A**) AlphaFold2-derived pLDDT scores for the top-ranked predictions of the N-terminal coiled-coil dimers of TRAK1 (top) and TRAK2 (bottom). Left: per-residue pLDDT plots for each coiled-coil prediction. Modelled residues with low confidence scores are highlighted using a grey bar, and are not displayed. Right: per-residue pLDDT prediction confidence mapped onto the predicted structures. For clarity, the coiled-coil is split into two separately aligned sections at the site of disorder around residue 200. (**B**) AlphaFold2-derived pLDDT scores for the top-ranked predictions of the N-terminal coiled-coil dimers of TRAK1 (top) and TRAK2 (bottom) in

complex with kinesin-1. Left: per-residue pLDDT plots for each complex prediction. Modelled residues with low confidence scores are highlighted using a grey bar, and are not displayed. Right: per-residue pLDDT prediction confidence mapped onto the predicted structures. For clarity, the coiled-coil is split into two separately aligned sections at the site of disorder around residue 200. (**C**) AlphaFold2-derived PAE plots for the top-ranked predictions of the N-terminal coiled-coil dimers of TRAK1 (left) and TRAK2 (right) in complex with kinesin-1. The N- and C- terminal portions of the structures displayed in (B) and Fig. 2A are indicated across the top of the plot.

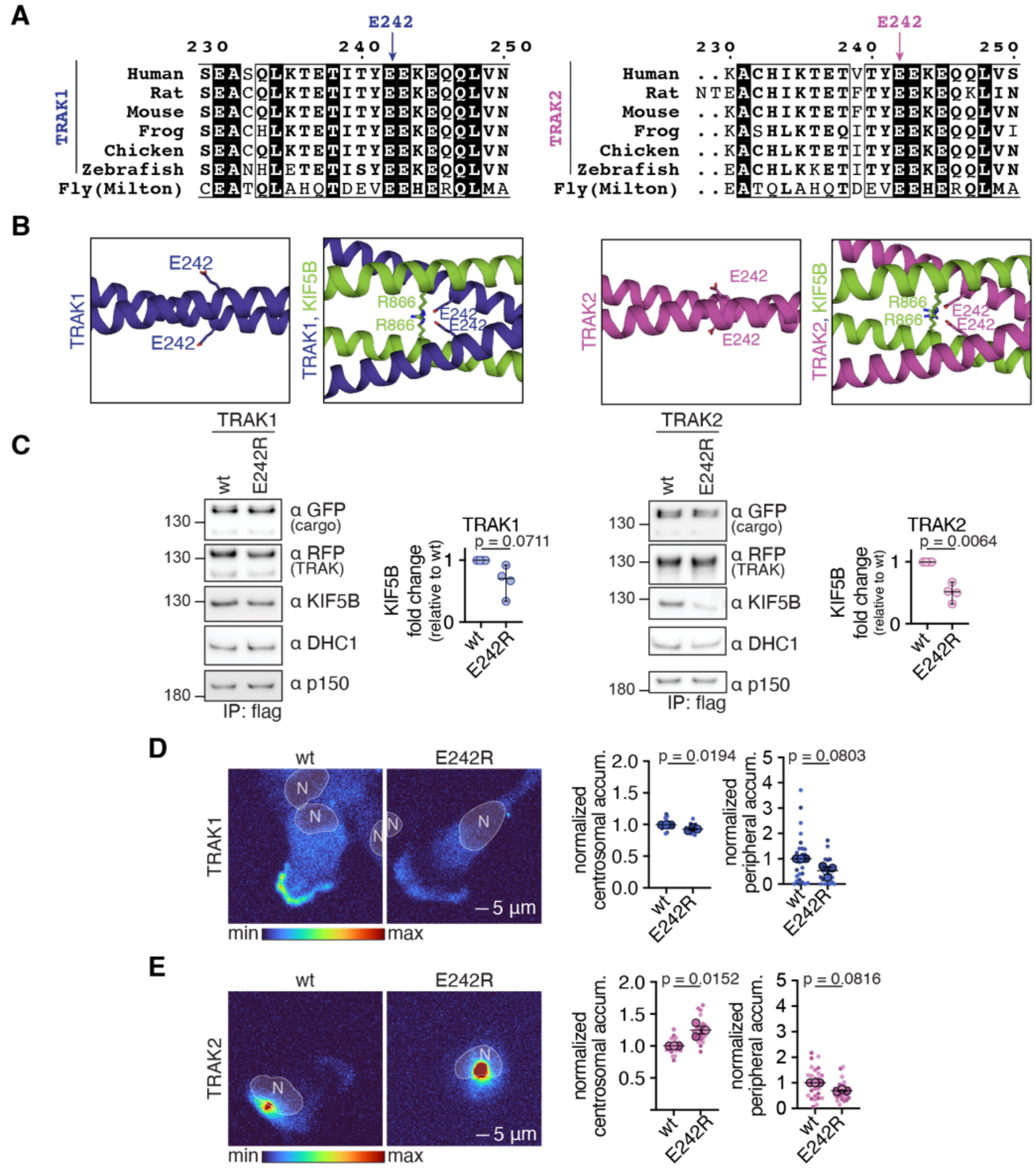

fig. S6. Validating the AlphaFold2 model for kinesin interaction.

(**A**) Sequence alignment showing E242 conservation in TRAK1 (left) and TRAK2 (right). (**B**) Insets from the TRAK1 (blue, left) and TRAK2 (purple, right) coiled-coil structure predictions with or without kinesin-1 bound (green). E242 residues in TRAK and R866 residues in kinesin-1 that are predicted to form a buried salt-bridge are highlighted. (**C**) Immunoblot analysis of the motor-adaptor components associated with FLAG-purified cargo from indicated cell lines. Quantification of relative motor protein levels normalized to wt. One-sample t-tests (n=4), p-values indicated in the figure. Error bars show the median and 95% confidence interval. (**D**), (**E**) Representative maximum intensity projection images and quantification of cargo accumulation from indicated cell lines. Nested t-test (n=3), p-values indicated in the figure. Error bars show the mean and SEM.

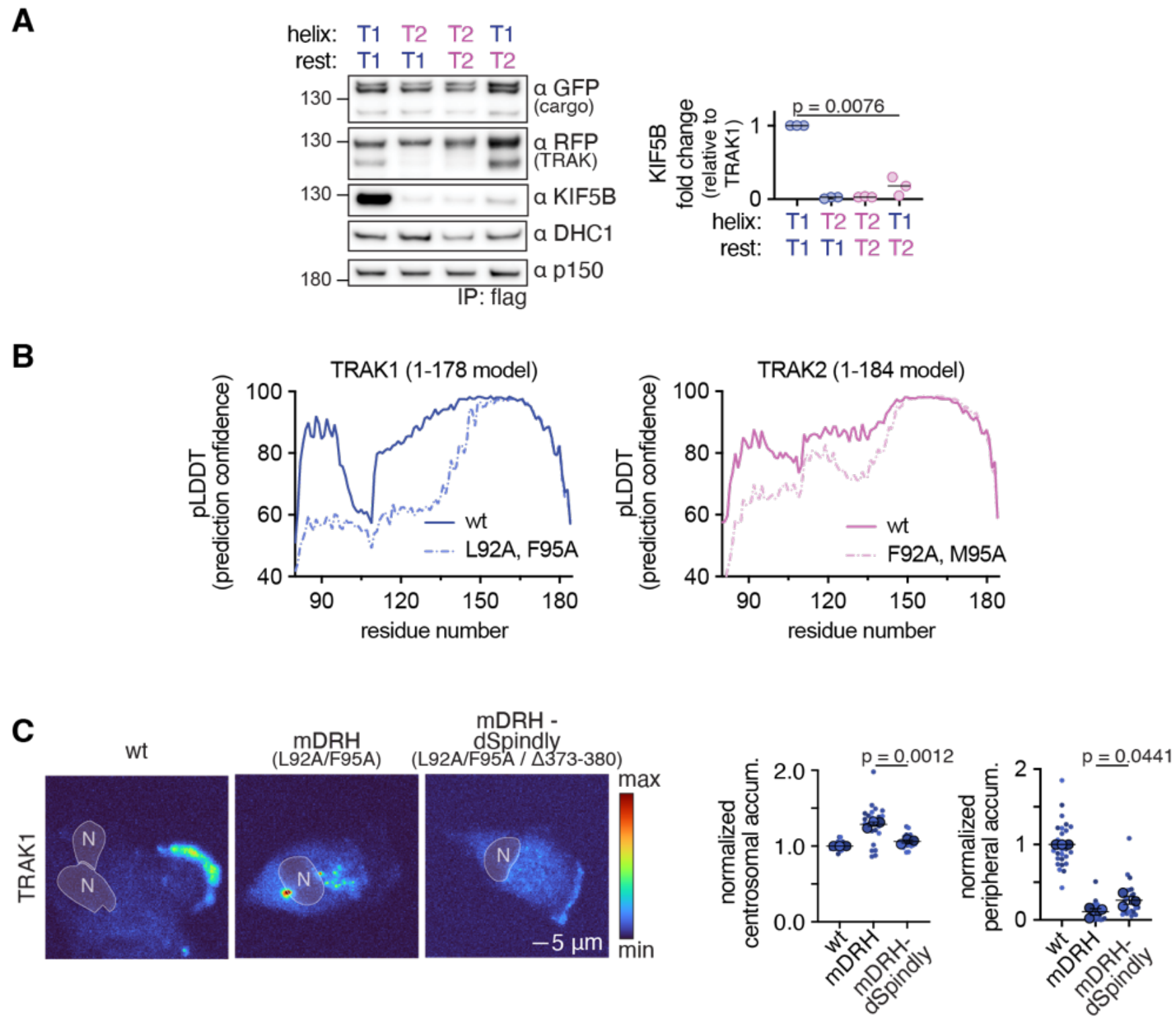

fig. S7. Testing the dynein regulation model.
(**A**) Left: Immunoblot analysis of the motor-adaptor components associated with FLAG-purified cargo from indicated cell lines. Right: quantification of relative motor protein levels normalized to wt. One-sample t-tests (n=3), p-values indicated in the figure. Error bars show the median and 95% confidence interval. (**B**) Per-residue AlphaFold2-derived pLDDT scores for the top-ranked predictions of the N-terminal coiled-coil dimers of TRAK1 (blue, left) and TRAK2 (purple, right). (**C**) Representative maximum intensity projection images and quantification of cargo accumulation from indicated cell lines. Nested ANOVA with Šídák's test (n=3), p-values indicated in the figure. Error bars show the mean and SEM.

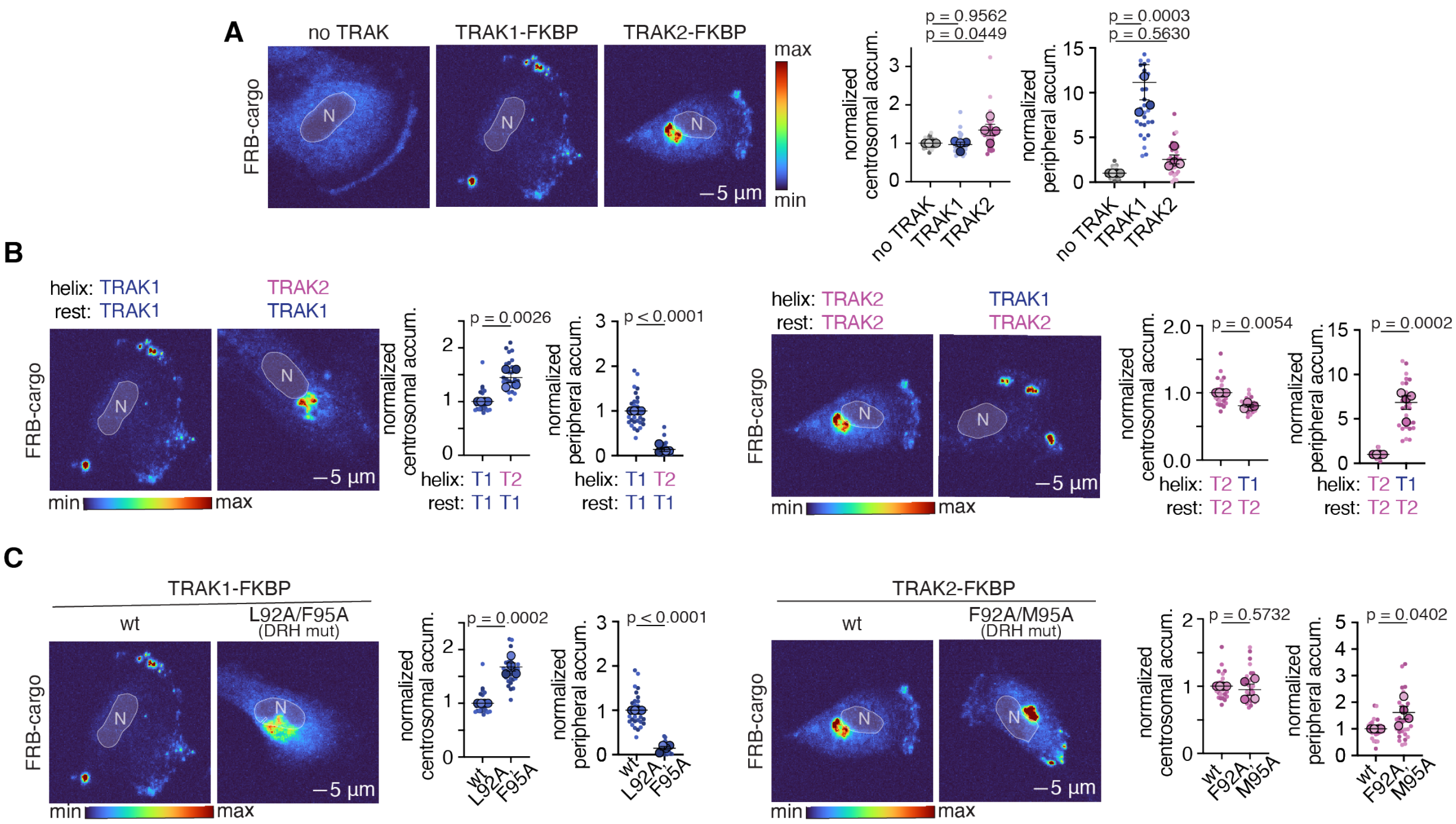

fig. S8. The dynein regulation model is independent of Miro1.
To test whether the proposed dynein regulatory model is dependent on Miro1, we linked TRAK variants directly to the synthetic cargo by using the FKBP-FRB inducible dimerization system. We compared the accumulation of cargo alone, and cargo decorated with TRAK1 or TRAK2 (**A**), investigated the effect of TRAK chimera proteins (**B**), and DRH disrupting mutants (**C**). (**A**), (**B**), (**C**) Representative maximum intensity projection images and quantification of cargo accumulation from indicated cell lines. Nested ANOVA with Dunnett's test (A) or nested t-test (B) and (C), (n=3), p-values indicated in the figure. Error bars show the mean and SEM.

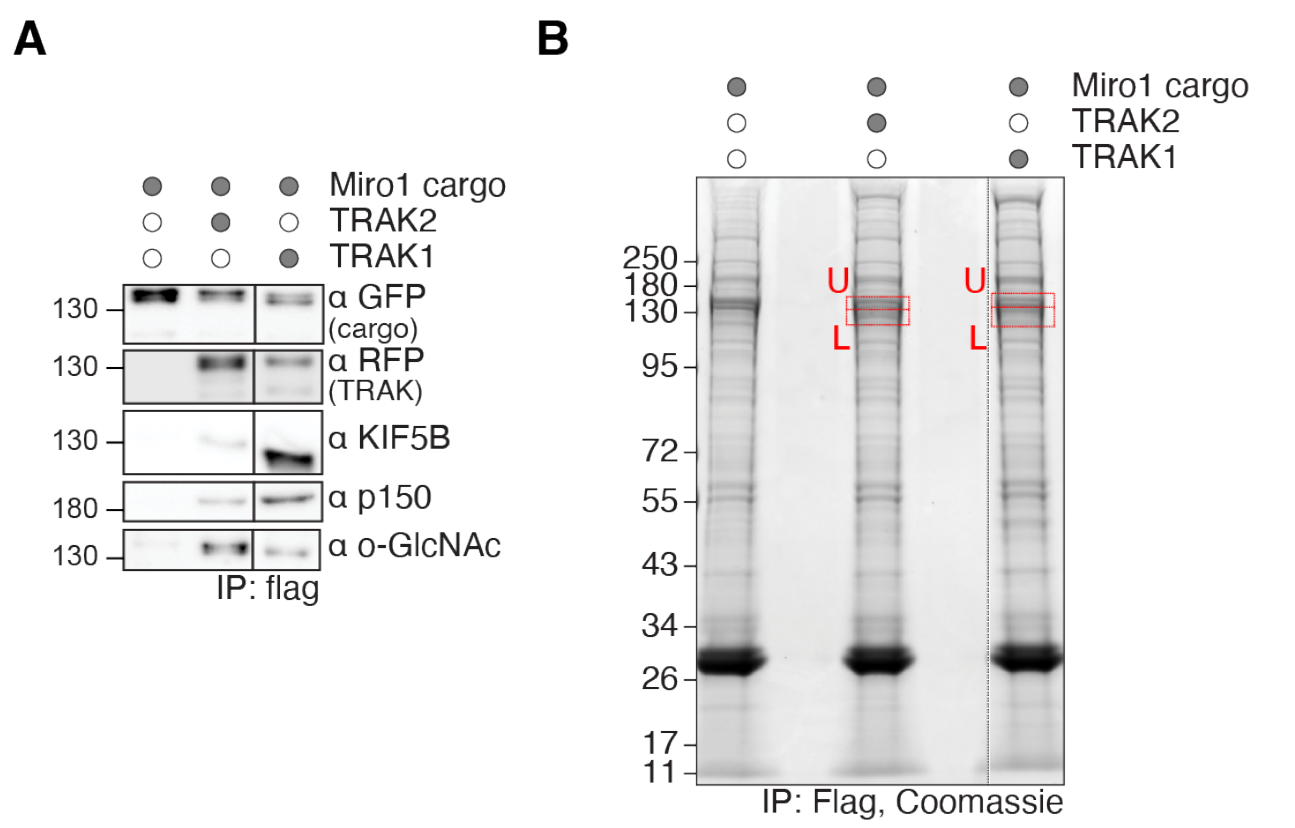

C

rank #	band: TRAK1 (U)	# Spectra	Coverage (%)
1	TRAK1	171	41.45 / 55.44
2	RHOT1 (Miro1)	329	56.11
3	KIF5B	187	59.81

rank #	band: TRAK1 (L)	# Spectra	Coverage (%)
1	TRAK1	320	52.99 / 70.87
2	KIF5B	372	74.56
3	OGT	170	56.41

rank #	band: TRAK2 (U)	# Spectra	Coverage (%)
1	TRAK2	471	48.14 / 62.85
2	IPO5	156	45.38
3	KIF5B	155	57.53

rank #	band: TRAK2 (L)	# Spectra	Coverage (%)
1	TRAK2	256	46.28 / 60.43
2	RHOT1 (Miro1)	352	52.76
3	SF3B2	101	43.69

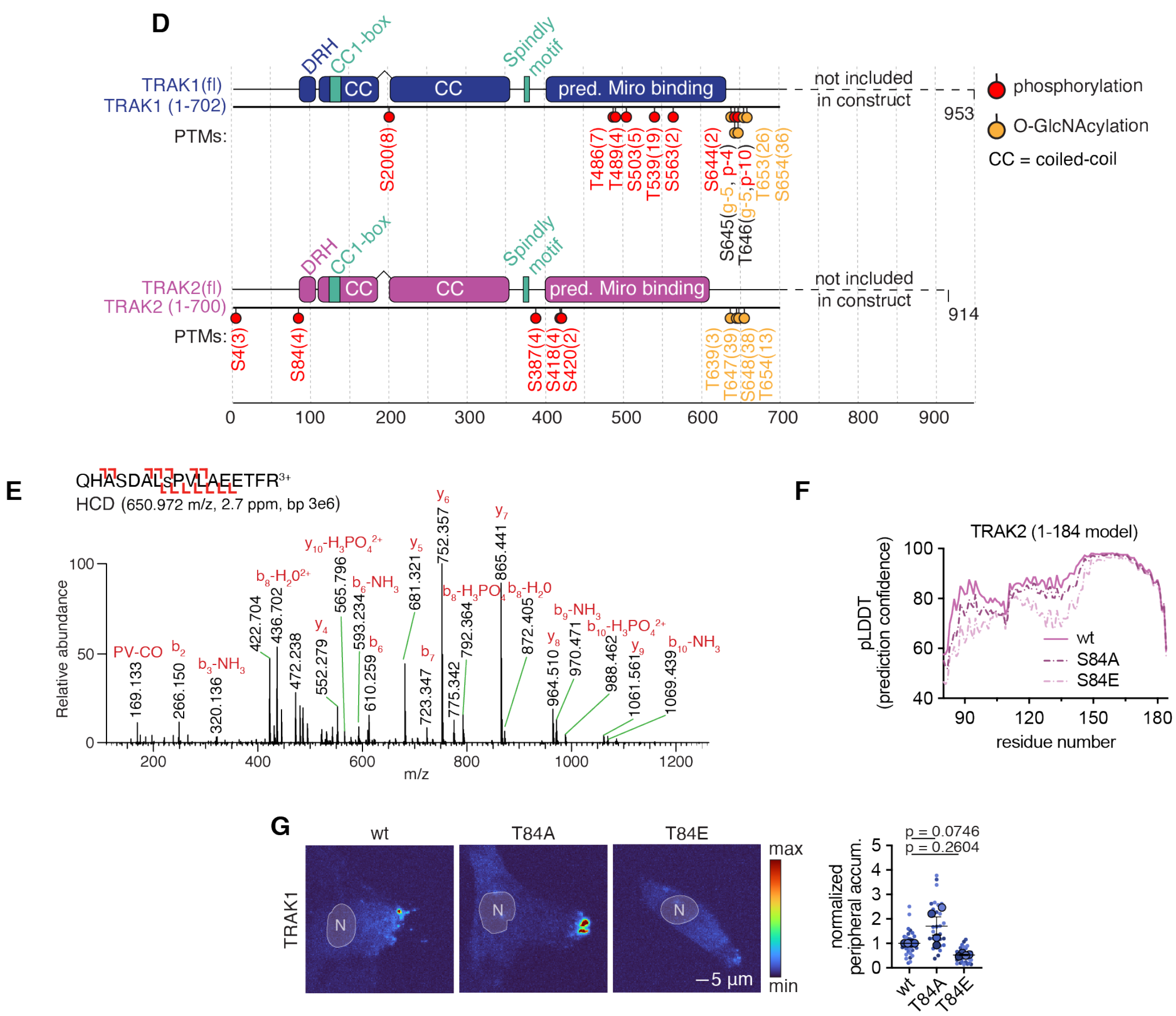

fig. S9. Mapping post-translational modifications of TRAK.

(**A**) Immunoblot analysis of the FLAG-purified cargo used for mass spectrometry. (**B**) SDS-PAGE separation of co-eluting proteins from purification analysed in (A). Bands cut for further protein and PTM analysis are highlighted in red. (**C**) Top ranked proteins identified in each sample. For TRAK, coverage is reported as % of full-length sequence / overexpressed construct. (**D**) PTM sites identified from bands cut in (B). Peptide spectra matches for each site are reported in brackets. (**E**) HCD mass spectrum for the TRAK2 phosphopeptide demonstrating modification of S84, in lower case. Observed m/z, mass error in ppm, and base peak (bp) is shown. The

TRAK2 S84 phosphorylation site was additionally found in several high-throughput studies (in PhosphoSitePlus (*59–61*), while no phosphorylation of T84 on TRAK1 has been reported (in PhosphoSitePlus). (**F**) Per-residue AlphaFold2-derived pLDDT scores for the top-ranked predictions of the N-terminal coiled-coil dimers of TRAK2 variants. (**G**) Representative maximum intensity projection images and quantification of cargo accumulation from indicated cell lines. Nested ANOVA with Dunnett's test (n=4), p-values indicated in the figure. Error bars show the mean and SEM.

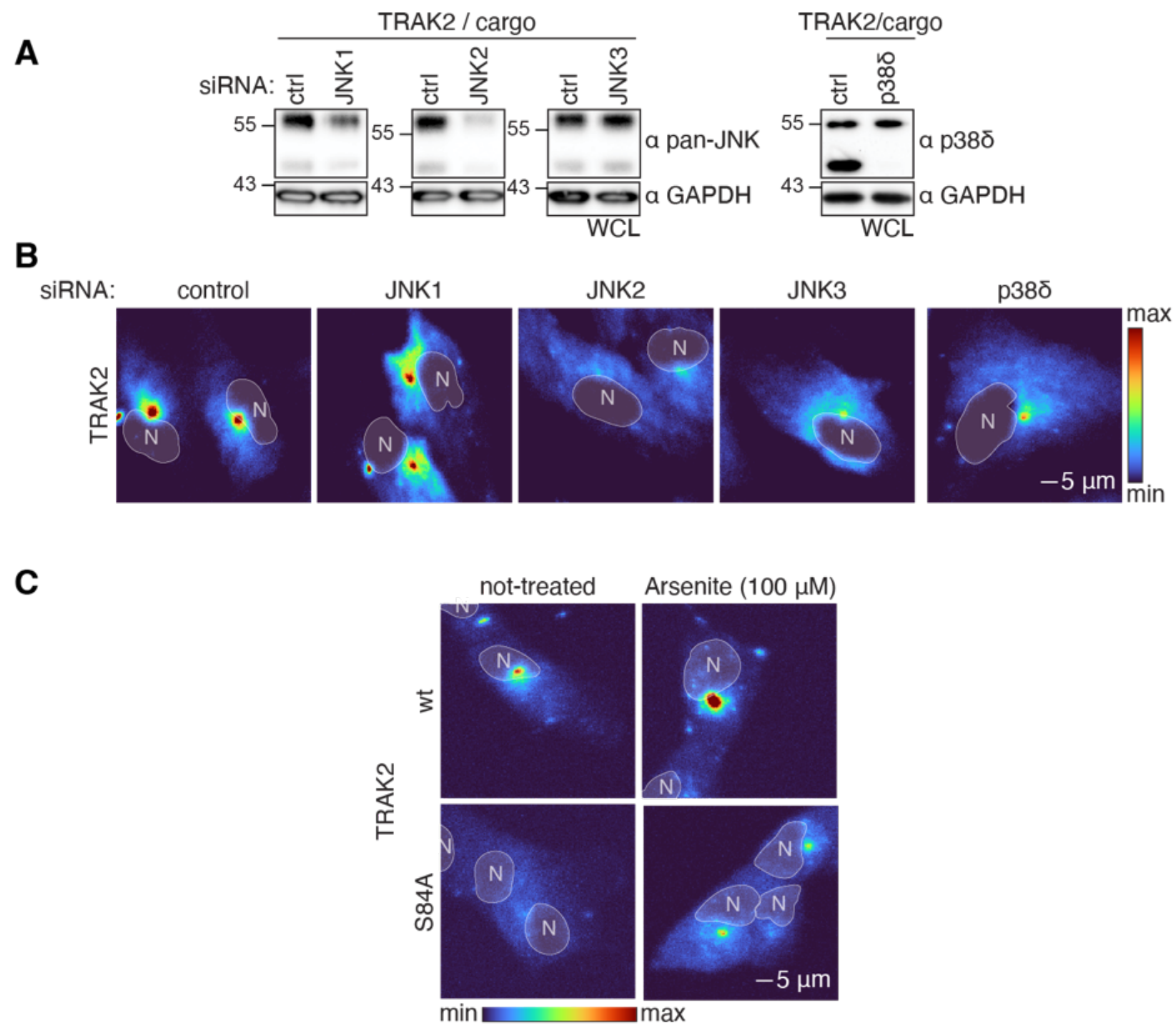

fig. S10. Characterizing Ser84 phosphorylation of TRAK2.

(**A**) Immunoblot analysis of whole cell lysate showing the efficacy of siRNA treatments used in Fig. 3E and Fig. 4F. (**B**) Representative maximum intensity projection images of cargo accumulation from TRAK2-expressing cells treated with indicated siRNAs. See Fig. 3E for quantification. (**C**) Representative maximum intensity projection images of cargo accumulation from TRAK2-expressing cells treated as indicated. See Fig. 3G for quantification.

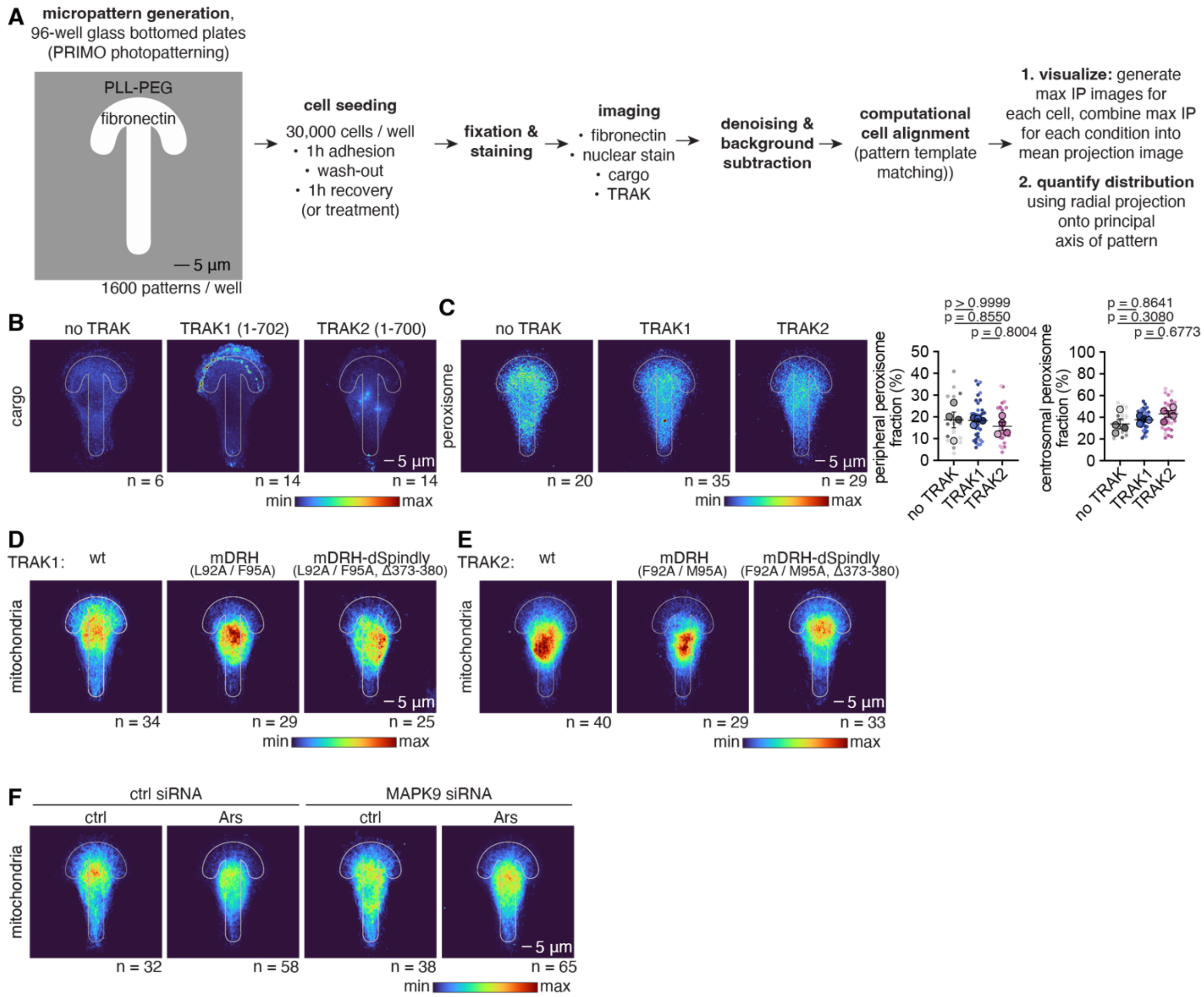

fig. S11. Measuring mitochondrial distribution in micropatterned cells.

(**A**) Measuring the distribution of mitochondria in cells grown in reproducible polarized shapes on a patterned fibronectin substrate (see Fig. 4B and Methods). (**B**) Mean projection images showing Miro1 synthetic cargo distribution in all cells measured for indicated cell lines. (**C**) Mean projection images showing peroxisome distribution in all cells measured for indicated cell lines. Quantification of the peripheral and centrosomal peroxisome fraction from indicated cell lines. Nested ANOVA with Šídák's test (n=4), p-values indicated in the figure. Error bars show the mean and SEM. (**D**), (**E**), (**F**) Mean projection images showing mitochondrial distribution in all cells measured for indicated cell lines.

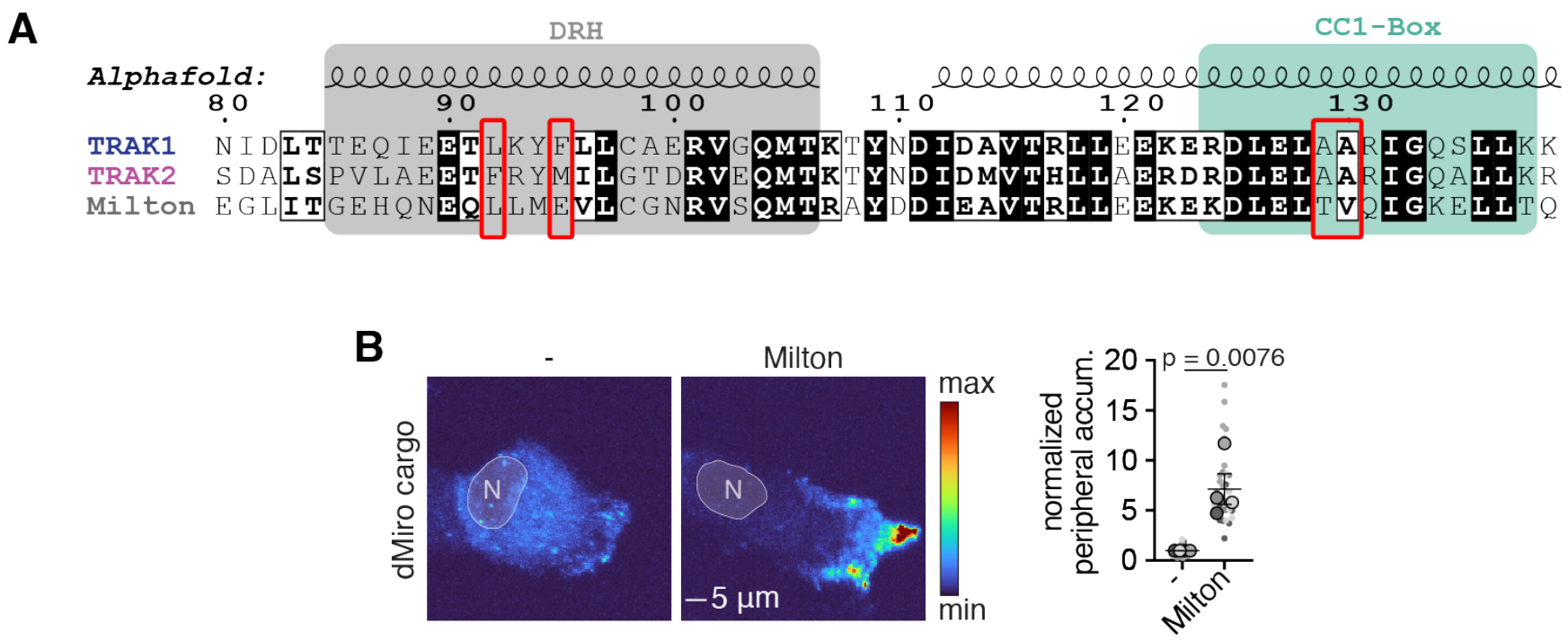

fig. S12. Transport of Milton-driven dMiro synthetic cargo in human cells.

(**A**) Sequence alignment of TRAK1, TRAK2 and Milton (the *Drosophila* TRAK orthologue) highlighting weak conservation of DRH dynein-LIC mimicking residues and CC1-box residues (red rectangles). (**B**) Representative maximum intensity projection images and quantification of cargo accumulation from indicated cell lines. Nested t-test (n=4), p-values indicated in the figure. Error bars show the mean and SEM.

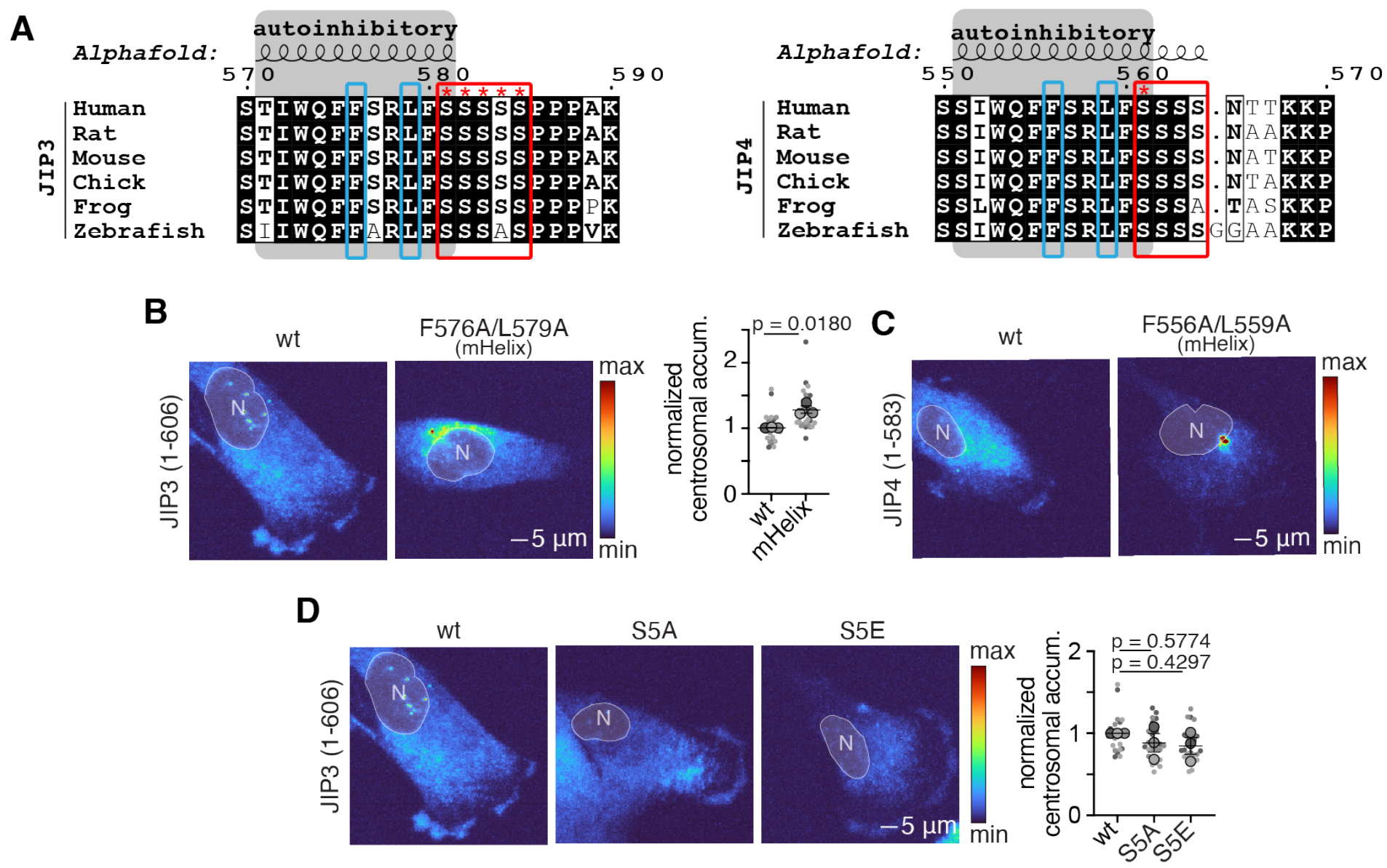

fig. S13. Regulation of JIP-mediated transport.

(**A**) Conservation of the autoinhibitory helix in JIP3 and 4 (*46*). Mapped secondary structure is based on AlphaFold3 prediction. Blue boxes highlight the conservation of dynein-LIC mimicking residues of the autoinhibitory helices, red boxes highlight flanking conserved serine residues. Stars indicate reportedly phosphorylated residues (*50*). (**B**) Representative images and quantification of the accumulation of cargo linked to indicated JIP3 constructs via the FKBP-FRB inducible dimerization system. Nested t-test (n=3), p-values indicated in the figure. Error bars show the mean and SEM. (**C**) Representative images of the accumulation of cargo linked to indicated JIP4 constructs via the FKBP-FRB inducible dimerization system. See Fig. 4G for quantification. (**D**) Representative images and quantification of the accumulation of cargo linked to indicated JIP3 constructs as in (B).

fig. S14 (below). Uncropped membrane images.
Source data corresponding to each immunoblot figure shown in the manuscript. Cropped membrane images are shown for input and reproduced for immunoprecipitated lanes. Overlays of chemiluminescent (signal) and colorimetric (ladder) images showing whole membranes probed with indicated primary and corresponding secondary antibodies. Cropped areas corresponding to input and IP lanes are highlighted with dashed rectangles. Lanes not shown in the study are indicated.

Fig. 1G

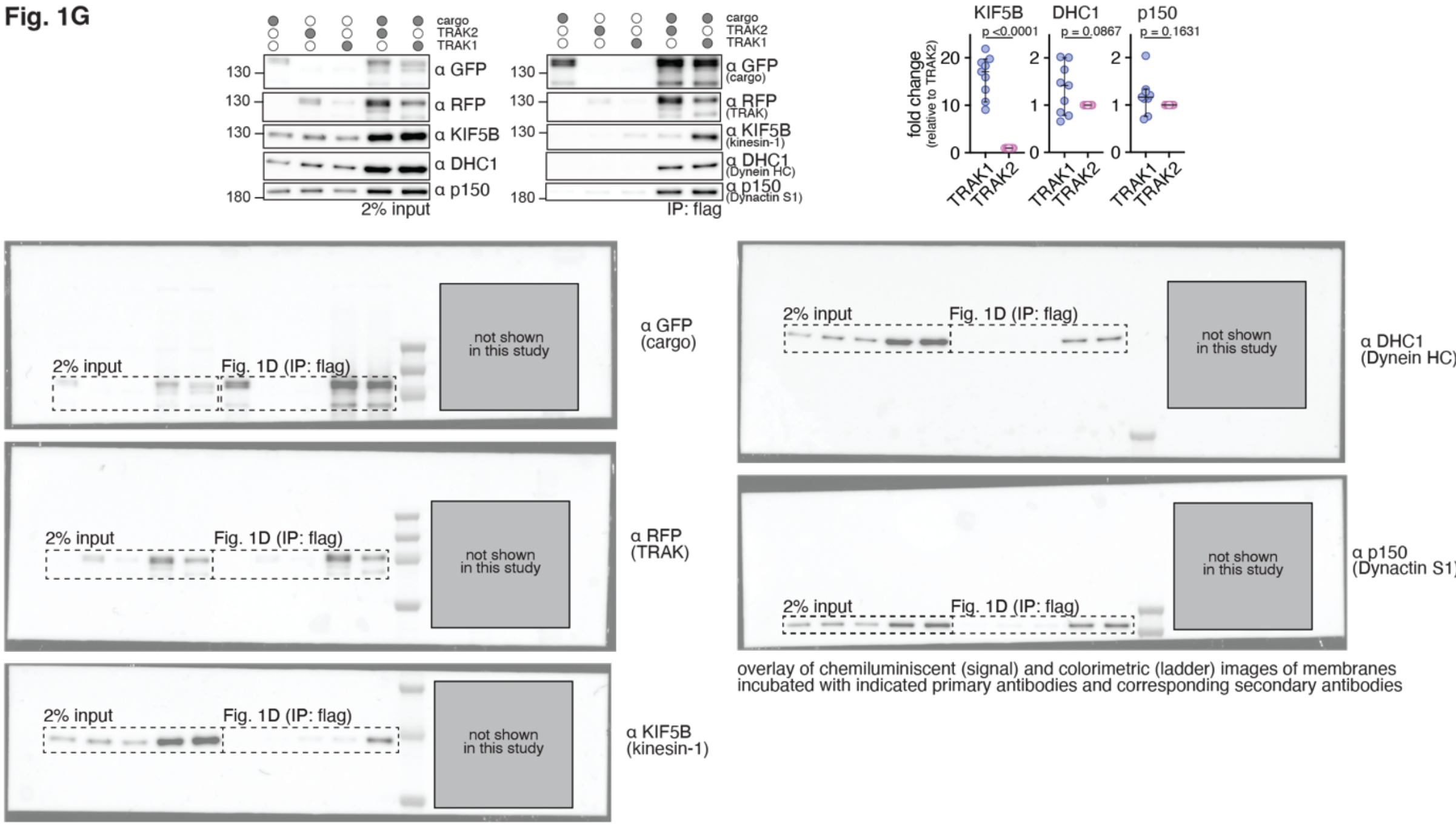

overlay of chemiluminiscent (signal) and colorimetric (ladder) images of membranes incubated with indicated primary antibodies and corresponding secondary antibodies

Fig. 1I

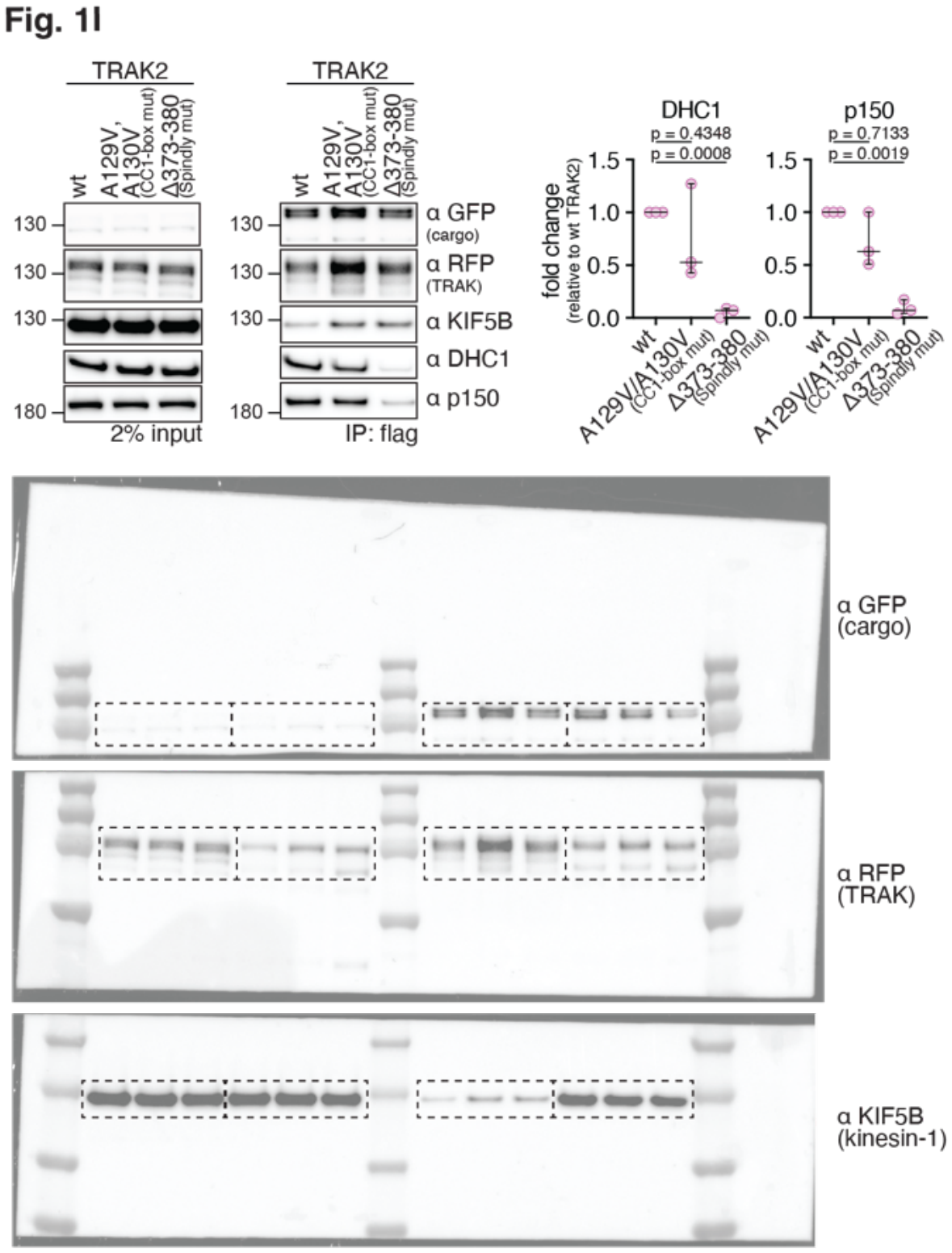

fig. S4H

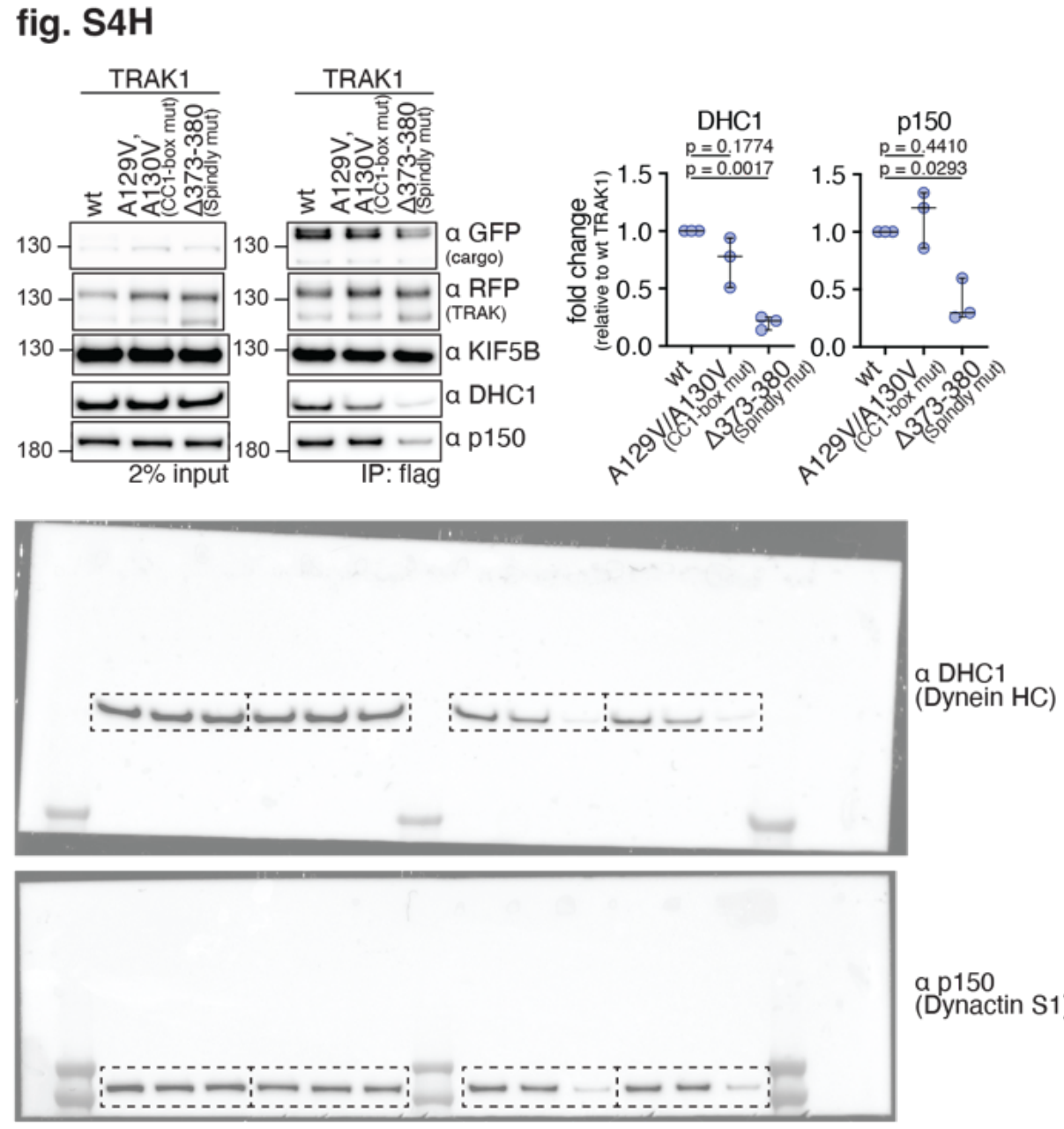

overlay of chemiluminiscent (signal) and colorimetric (ladder) images of membranes incubated with indicated primary antibodies and corresponding secondary antibodies

Fig. 2G

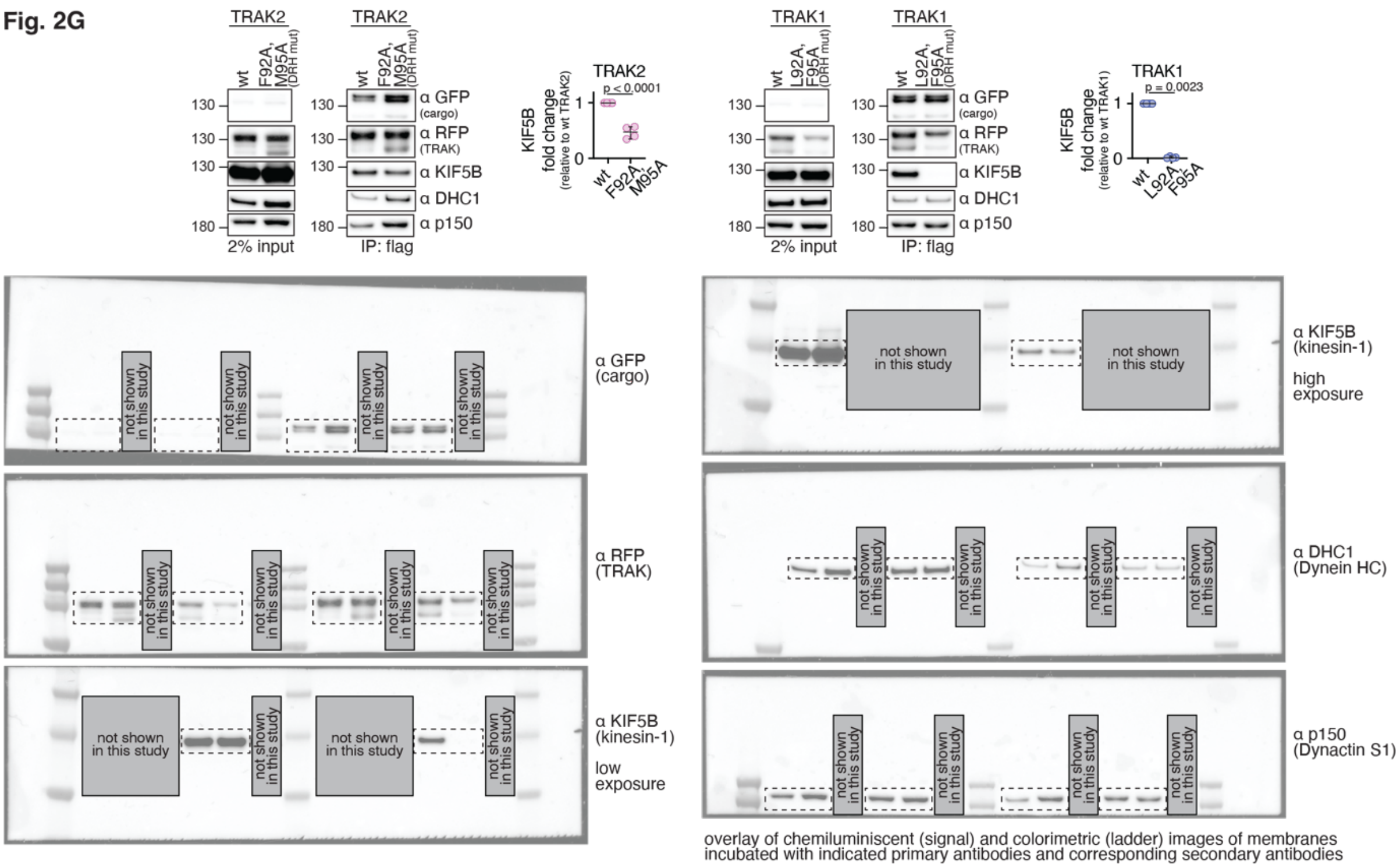
TRAK2
wt
F92A, M95A (DRH mut)
α GFP (cargo)
α RFP (TRAK)
α KIF5B
α DHC1
α p150
130
180
2% input
IP: flag
KIF5B fold change (relative to wt TRAK2)
p < 0.0001
TRAK1
L92A, F95A (DRH mut)
KIF5B fold change (relative to wt TRAK1)
p = 0.0023
not shown in this study
α KIF5B (kinesin-1) high exposure
α KIF5B (kinesin-1) low exposure
α DHC1 (Dynein HC)
α p150 (Dynactin S1)
overlay of chemiluminiscent (signal) and colorimetric (ladder) images of membranes incubated with indicated primary antibodies and corresponding secondary antibodies

Fig. 2H

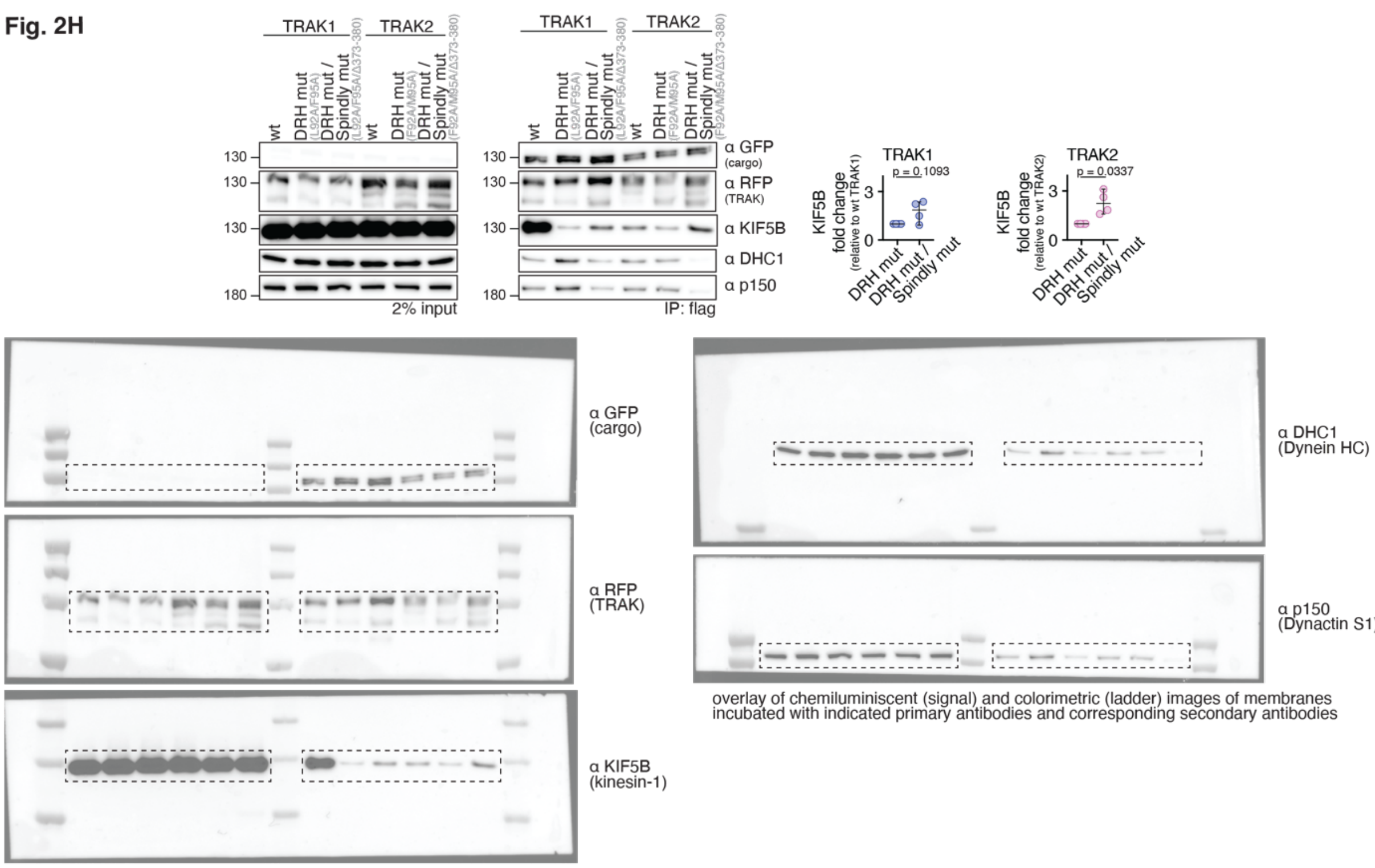
TRAK1
TRAK2
wt
DRH mut (L92A/F95A)
DRH mut / Spindly mut (L92A/F95A/Δ373-380)
DRH mut (F92A/M95A)
DRH mut / Spindly mut (F92A/M95A/Δ373-380)
α GFP (cargo)
α RFP (TRAK)
α KIF5B
α DHC1
α p150
2% input
IP: flag
KIF5B fold change (relative to wt TRAK1)
p = 0.1093
KIF5B fold change (relative to wt TRAK2)
p = 0.0337
α KIF5B (kinesin-1)
α DHC1 (Dynein HC)
α p150 (Dynactin S1)
overlay of chemiluminiscent (signal) and colorimetric (ladder) images of membranes incubated with indicated primary antibodies and corresponding secondary antibodies

Fig. 3D

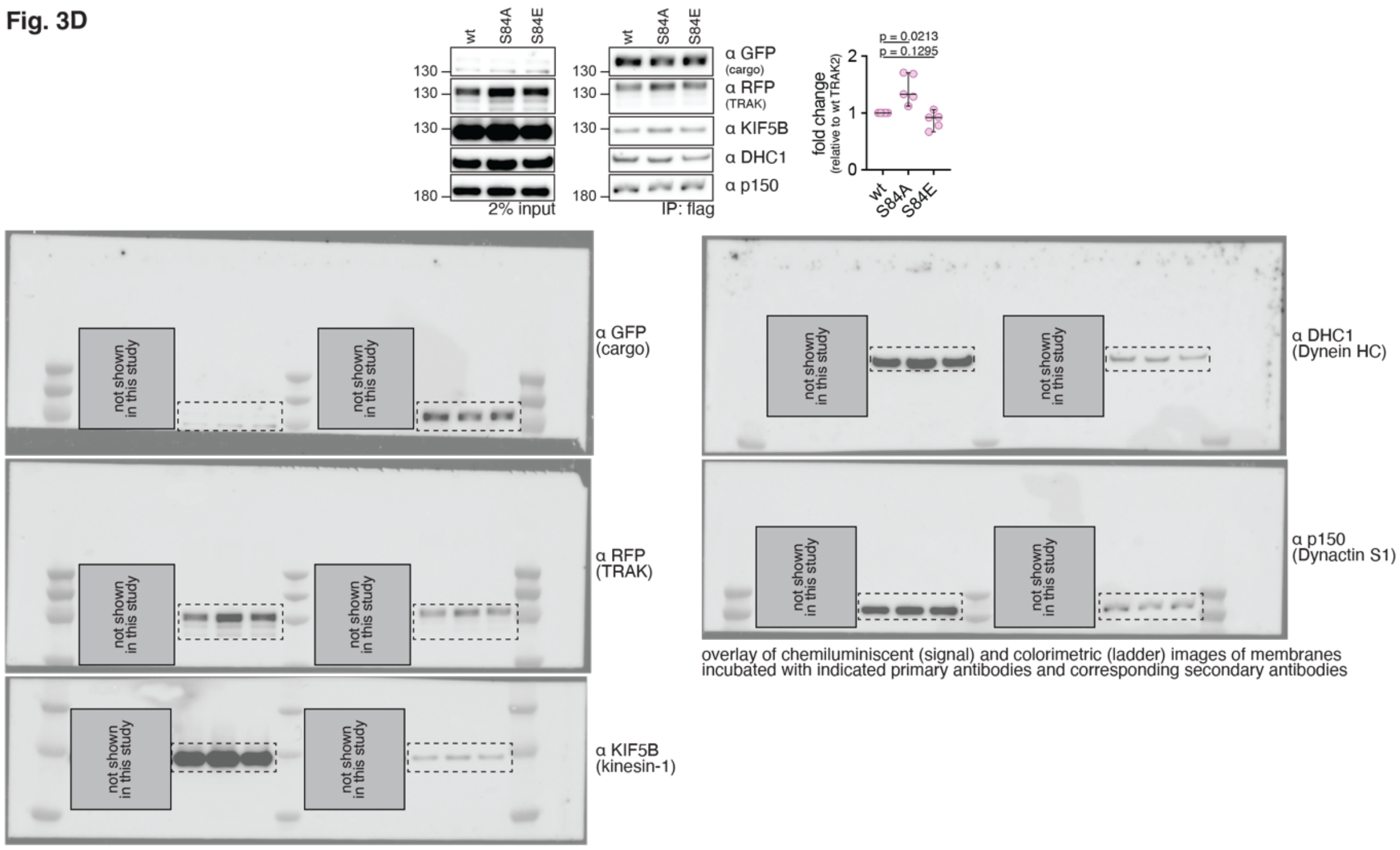

wt
S84A
S84E
130
180
2% input
IP: flag
α GFP (cargo)
α RFP (TRAK)
α KIF5B
α DHC1
α p150
p = 0.0213
p = 0.1295
fold change (relative to wt TRAK2)
not shown in this study
α GFP (cargo)
α RFP (TRAK)
α KIF5B (kinesin-1)
α DHC1 (Dynein HC)
α p150 (Dynactin S1)
overlay of chemiluminiscent (signal) and colorimetric (ladder) images of membranes incubated with indicated primary antibodies and corresponding secondary antibodies

Fig. 3F

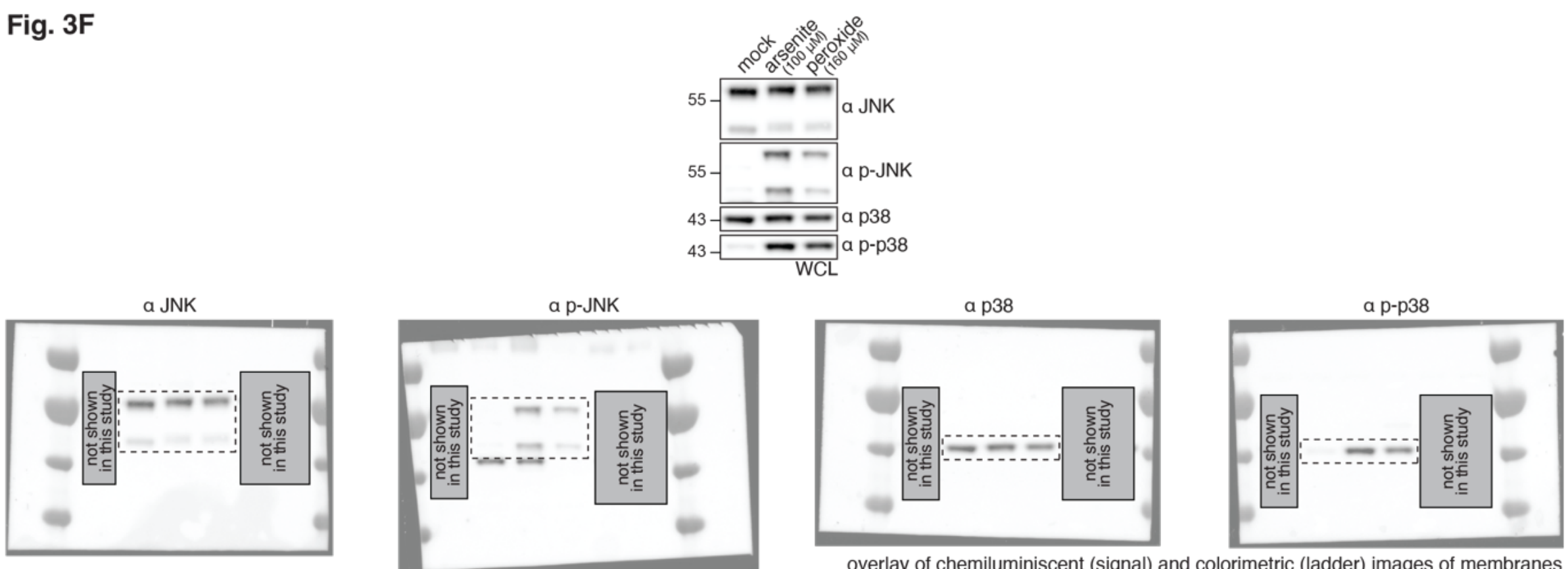

mock
arsenite (100 µM)
peroxide (160 µM)
55
43
α JNK
α p-JNK
α p38
α p-p38
WCL
not shown in this study
overlay of chemiluminiscent (signal) and colorimetric (ladder) images of membranes incubated with indicated primary antibodies and corresponding secondary antibodies

Fig. S3A

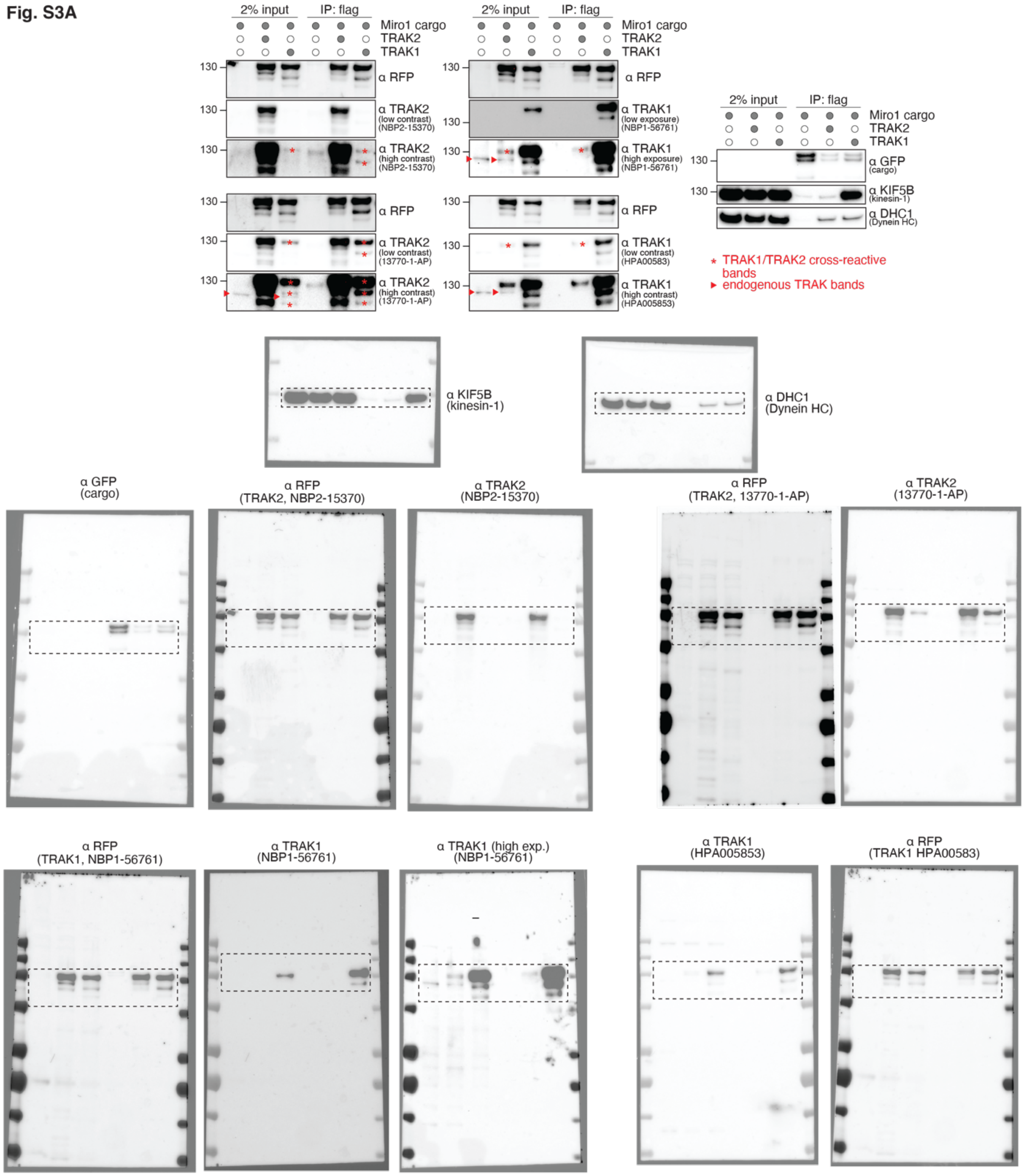
2% input
IP: flag
Miro1 cargo
TRAK2
TRAK1
130
α RFP
α TRAK2 (low contrast) (NBP2-15370)
α TRAK2 (high contrast) (NBP2-15370)
α TRAK2 (low contrast) (13770-1-AP)
α TRAK2 (high contrast) (13770-1-AP)
α TRAK1 (low exposure) (NBP1-56761)
α TRAK1 (high exposure) (NBP1-56761)
α TRAK1 (low contrast) (HPA00583)
α TRAK1 (high contrast) (HPA005853)
α GFP (cargo)
α KIF5B (kinesin-1)
α DHC1 (Dynein HC)
* TRAK1/TRAK2 cross-reactive bands
▸ endogenous TRAK bands
α KIF5B (kinesin-1)
α DHC1 (Dynein HC)
α GFP (cargo)
α RFP (TRAK2, NBP2-15370)
α TRAK2 (NBP2-15370)
α RFP (TRAK2, 13770-1-AP)
α TRAK2 (13770-1-AP)
α RFP (TRAK1, NBP1-56761)
α TRAK1 (NBP1-56761)
α TRAK1 (high exp.) (NBP1-56761)
α TRAK1 (HPA005853)
α RFP (TRAK1 HPA00583)

Fig. S4A-D

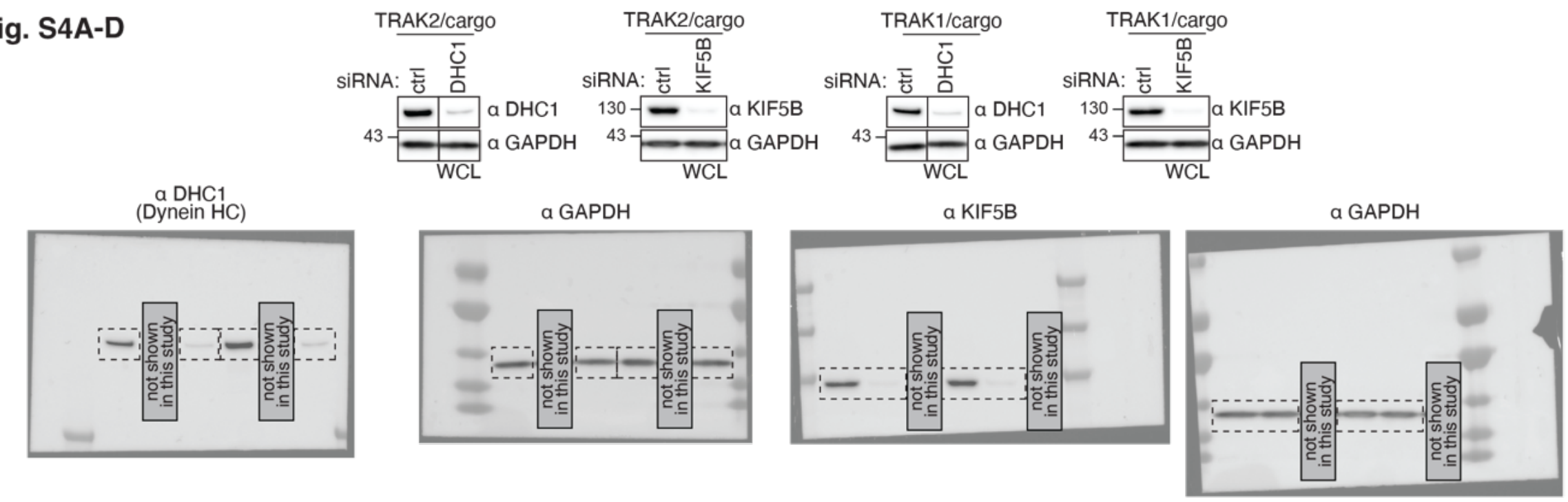

Fig. S4E

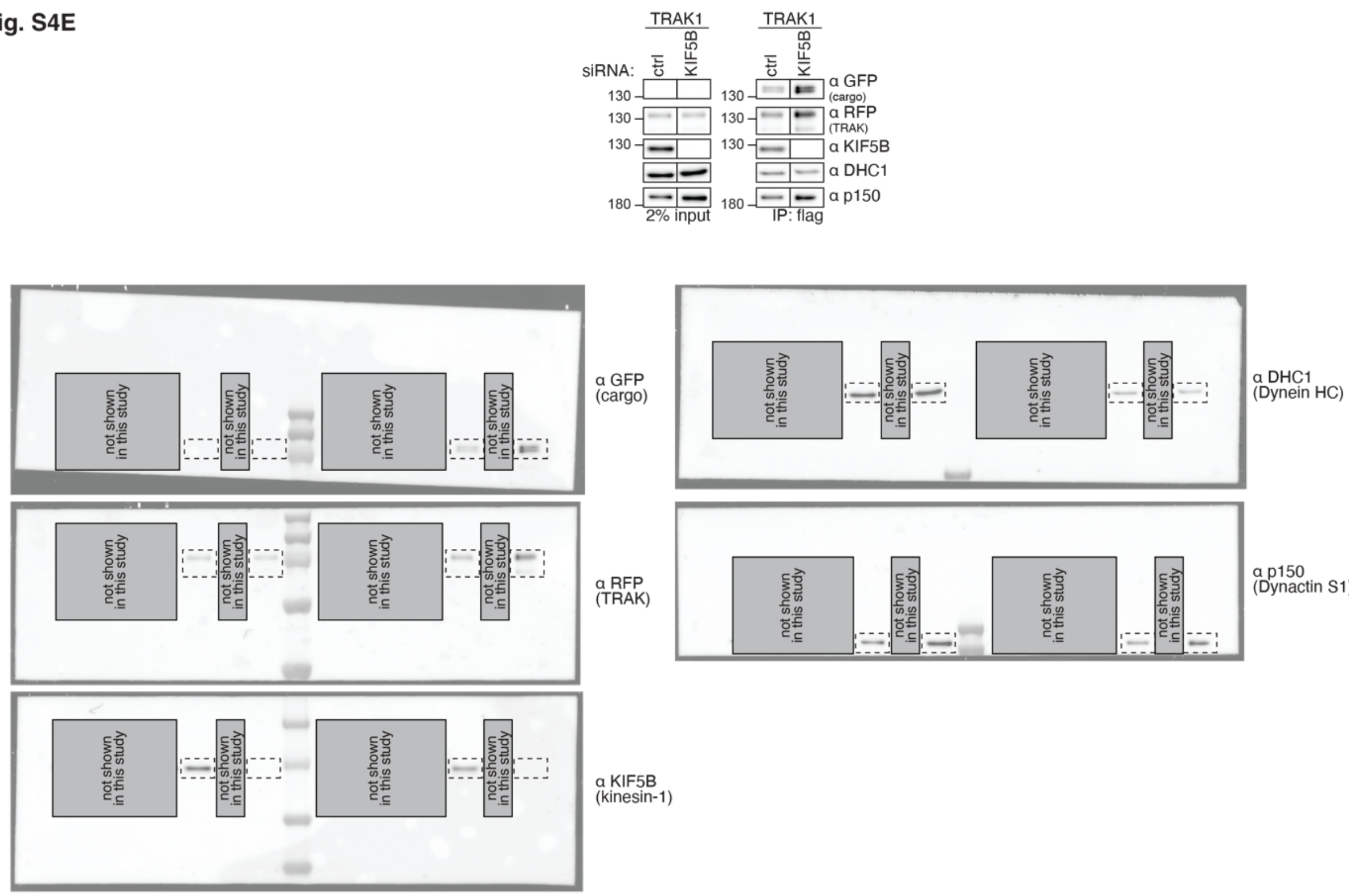

Fig. S6C (left)

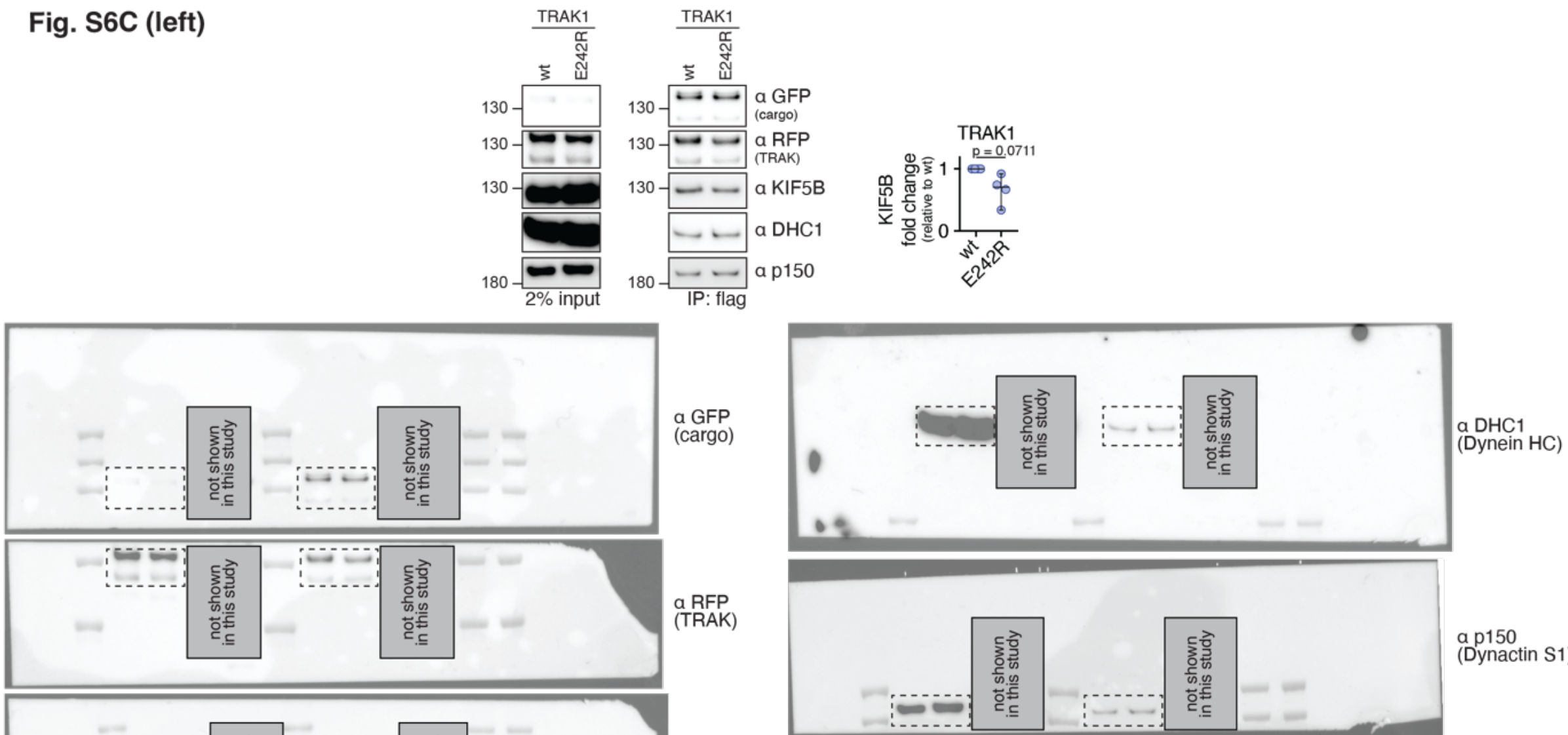

overlay of chemiluminiscent (signal) and colorimetric (ladder) images of membranes incubated with indicated primary antibodies and corresponding secondary antibodies

Fig. S6C (right)

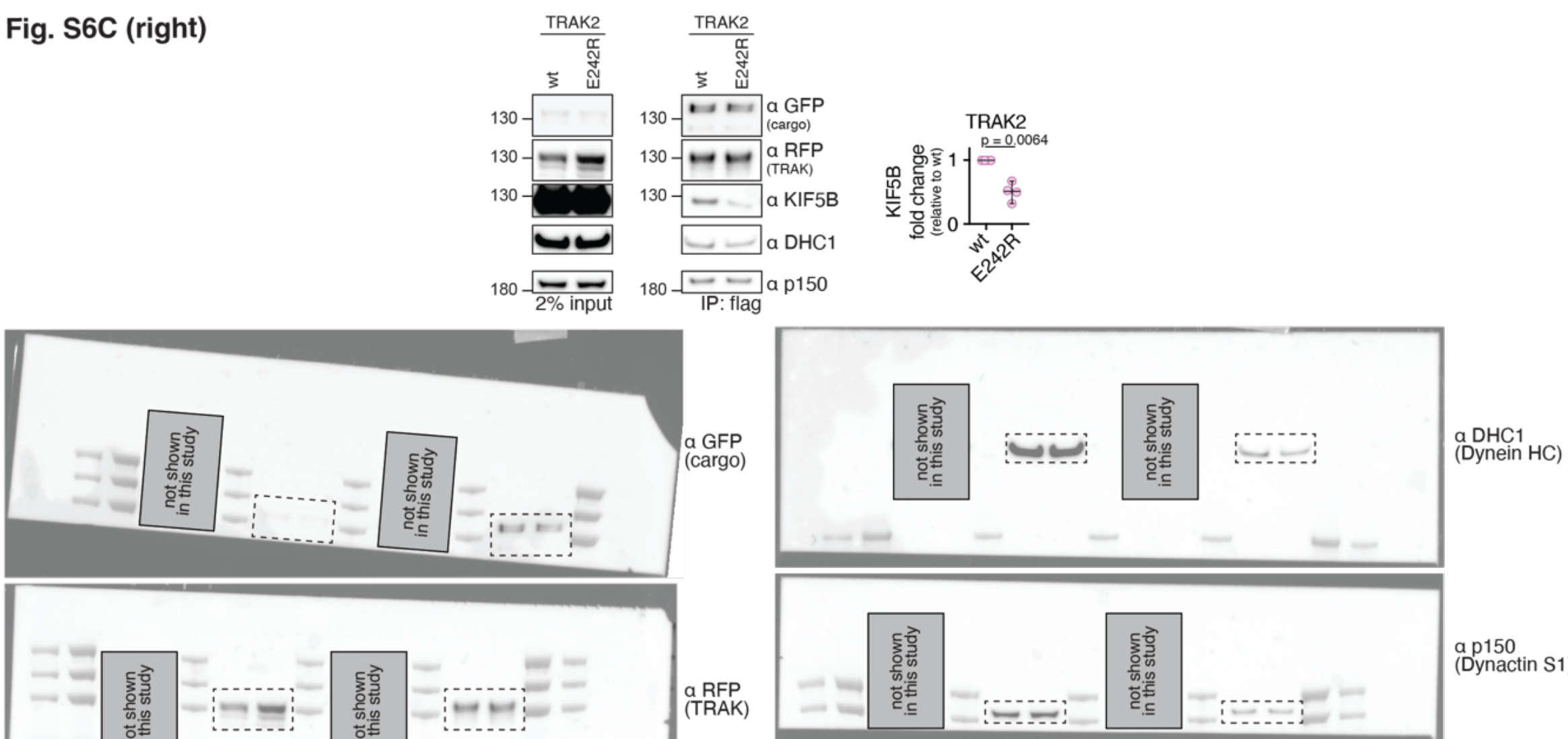

overlay of chemiluminiscent (signal) and colorimetric (ladder) images of membranes incubated with indicated primary antibodies and corresponding secondary antibodies

Fig. S7A

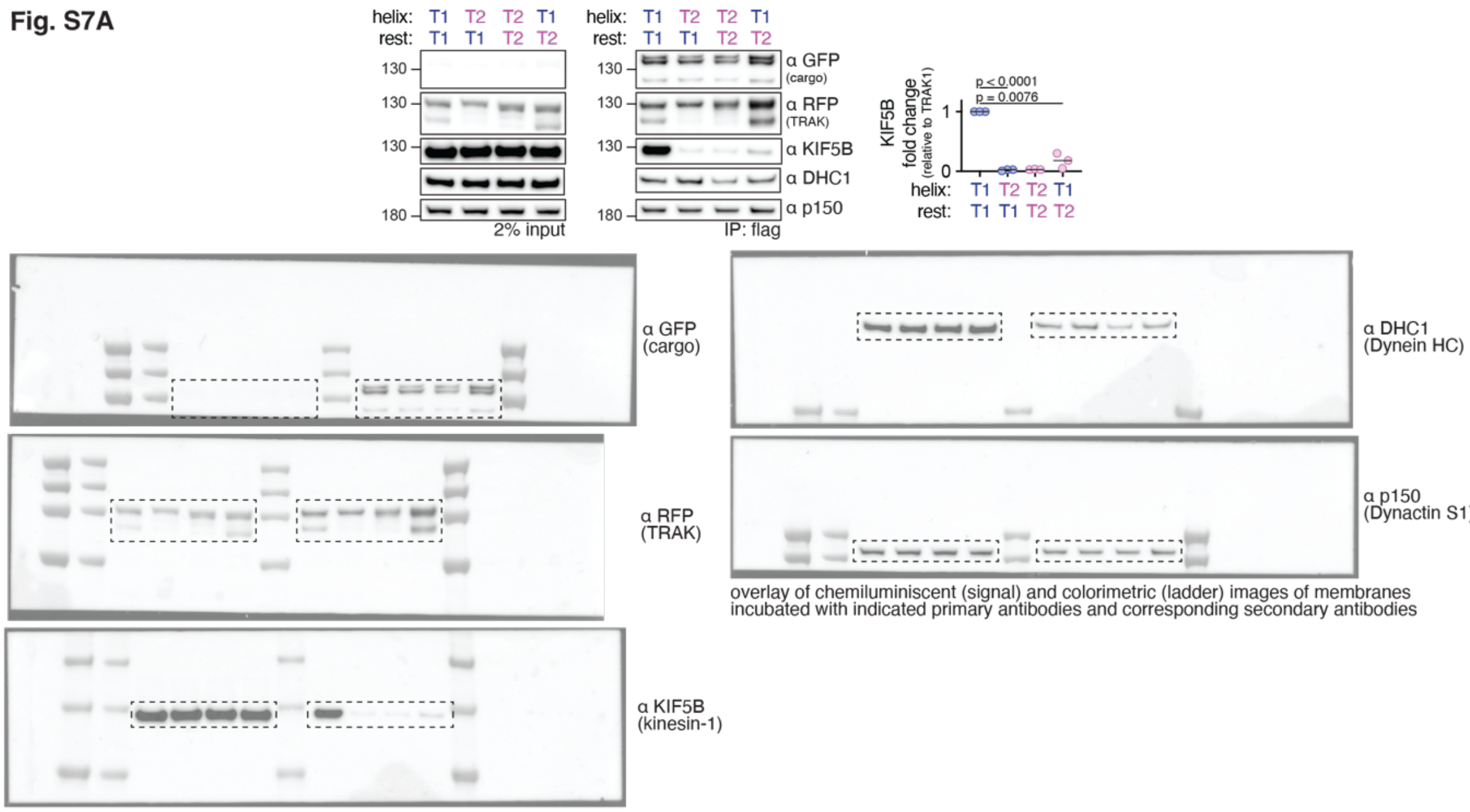

Fig. S9A

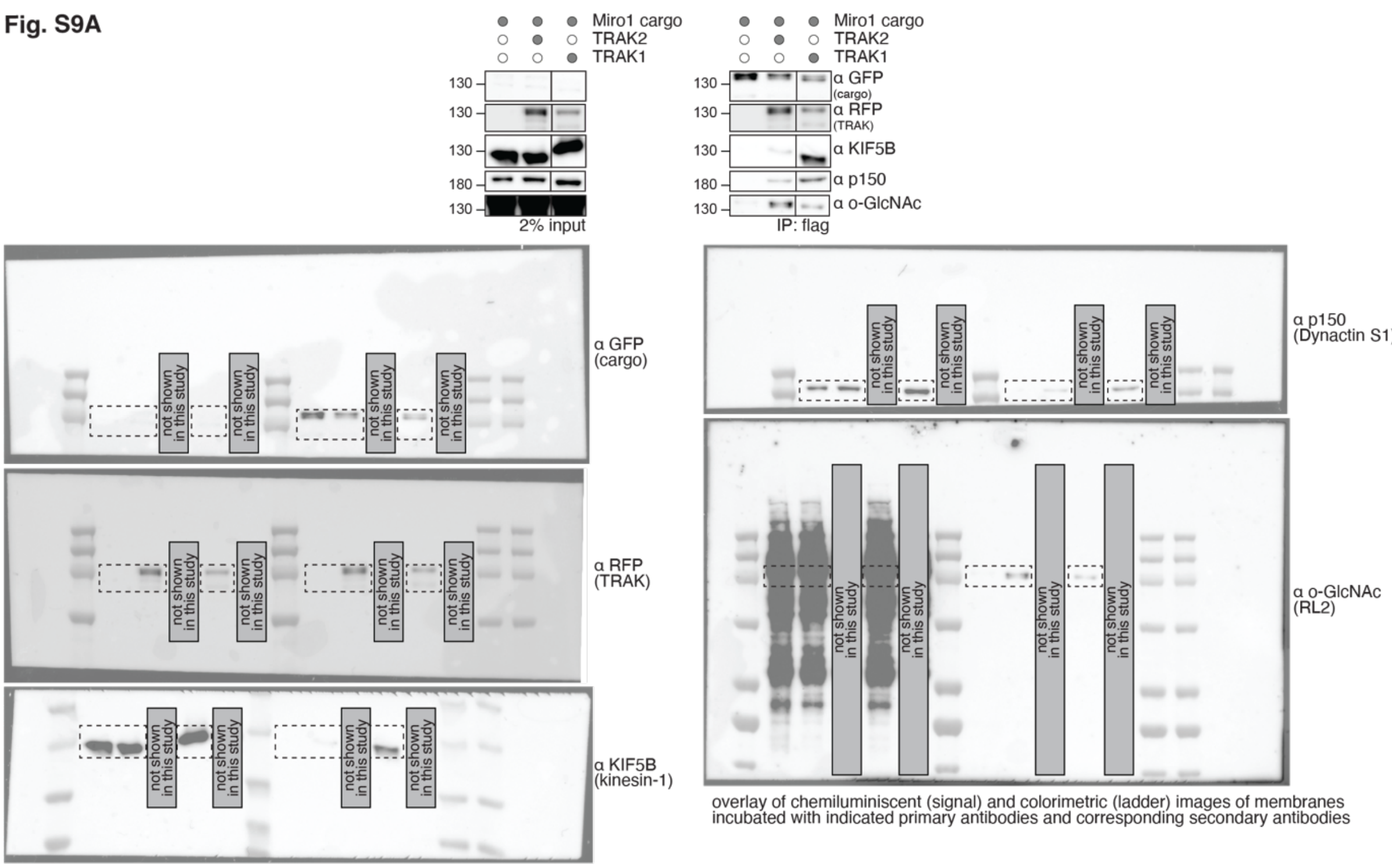

Fig. S10

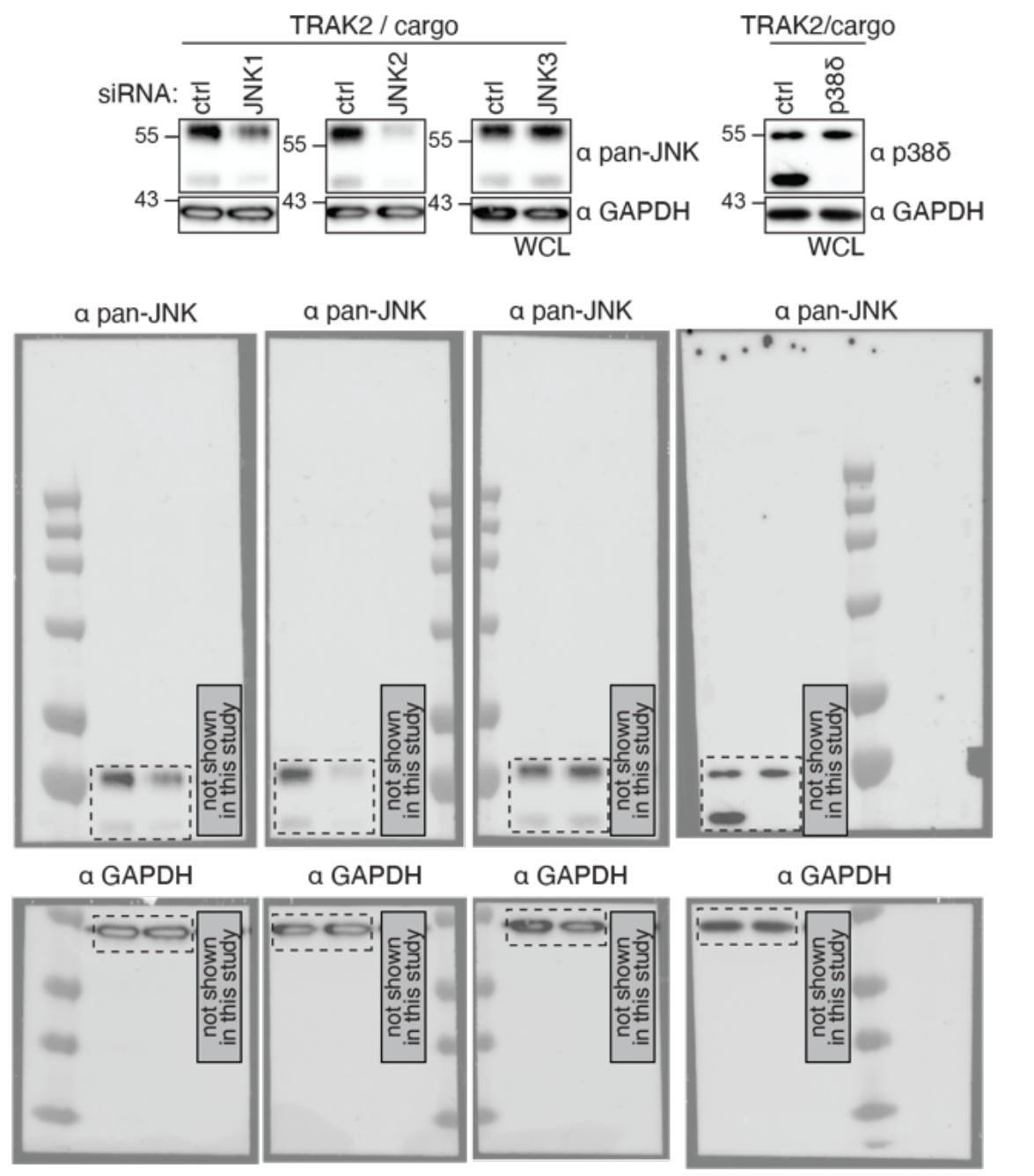

Methods

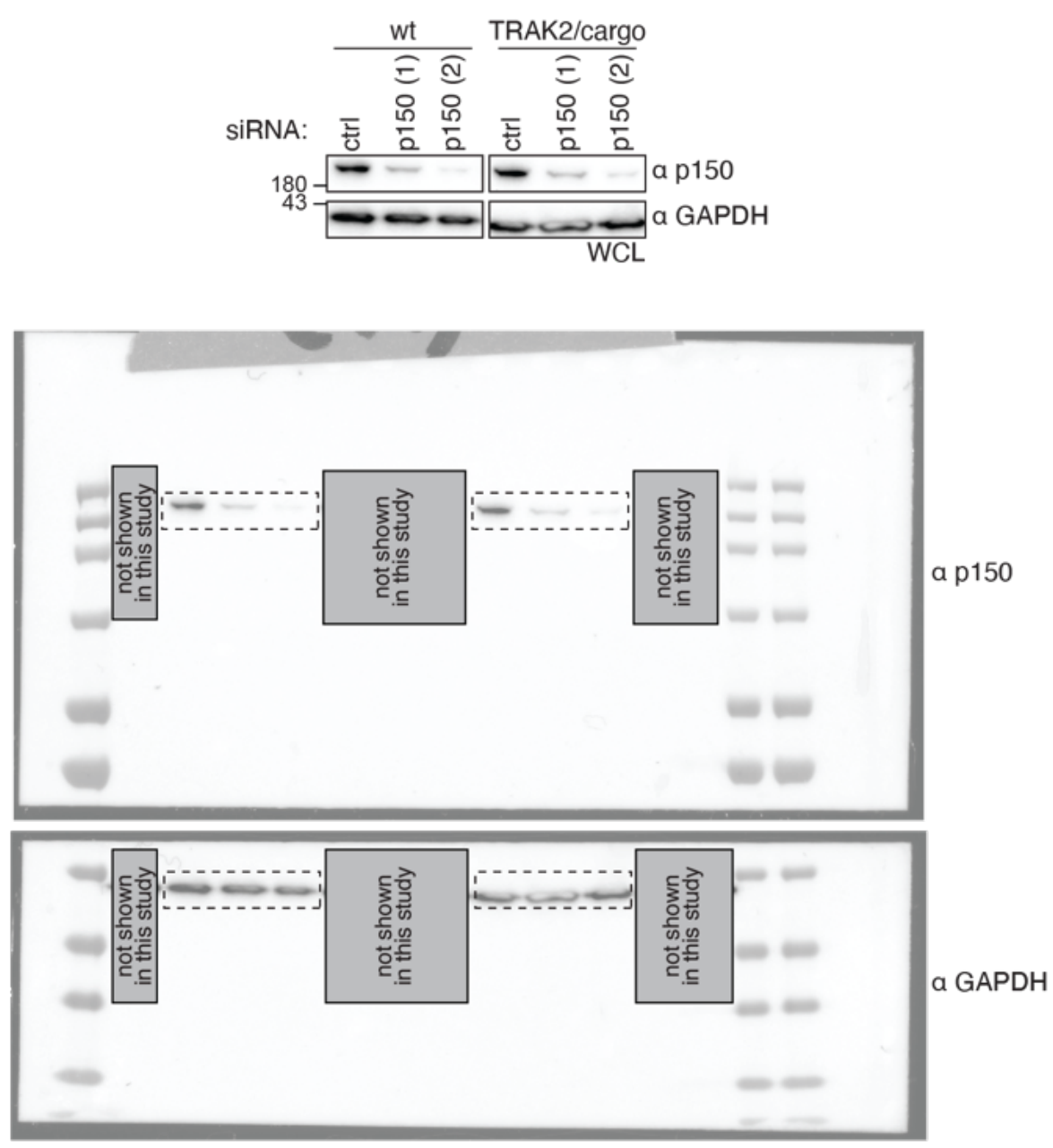

Movie S1. Synthetic cargo accumulation is microtubule dependent.
Reduction in accumulation of (A) TRAK1- and (B) TRAK2- driven synthetic cargo following microtubule disassembly by nocodazole treatment (2.5 µM, 30 min), and re-accumulation following microtubule re-assembly mediated by nocodazole wash-out. Scale bar = 5 µm. Time = mm:ss.

Movie S2. Synthetic cargo dynamics.
Dynamics of PA JF-646-labelled cargo after photoactivation of synthetic cargo in the absence of TRAK co-expression (A), TRAK1 co-expression (B), TRAK2 co-expression (C). The video for each condition is preceded by a still image showing cell orientation. Photoactivatable JF-646-labelled cargo dynamics mediated by TRAK expression as indicated. Scale bar = 5 µm. Time = mm:ss.
